# Nasopharyngeal Carriage Rate, Risk Factors, and Co‐Resistance Patterns of Methicillin‐Resistant *Staphylococcus aureus* in Ethiopia: Systematic Review and Meta‐Analysis

**DOI:** 10.1002/mbo3.70409

**Published:** 2026-09-09

**Authors:** Simachew Getaneh Endalamew, Sofiya Ayalew Kebede, Solomon Keflie Assefa, Adem Tsegaw Zegeye, Belayneh Jejaw Abate, Dejen kahsay Asgedom, Eliyas Addisu Taye, Endalew Minwuye Andargie, Eyob Akalewold Alemu, Halima Ayalew Kebede, Alebachew Tilahun Wassie, Andnet Yirga Assefa, Yihenew Getahun Ambaw, Helen Brhan Alemaw

**Affiliations:** ^1^ Department of Veterinary Epidemiology and Public Health, School of Veterinary Medicine Bahir Dar University Bahir Dar Ethiopia; ^2^ Department of Epidemiology and Biostatistics, School of Public Health, College of Medicine and Health Sciences Wollo University Dessie Ethiopia; ^3^ Department of Public Health Pawi Health Science College Pawi Ethiopia; ^4^ Department of Health Informatics, College of Medicine and Health Sciences University of Gondar Comprehensive Specialized Hospital Gondar Ethiopia; ^5^ University of Gondar Comprehensive Specialized Hospital Gondar Ethiopia; ^6^ Department of Public Health, College of Medicine and Health Science Samara University Samara Ethiopia; ^7^ Department of Public Health, School of Public Health, Asrat Woldeyes Health Science Campus Debre Berhan University Debre Berhan Ethiopia; ^8^ Department of Public Health, Institute of Public Health, College of Medicine and Health Sciences University of Gondar Gondar Ethiopia; ^9^ Department of Public Health, College of Health Sciences Woldia University Woldia Ethiopia; ^10^ Department of Clinical Medicine, School of Veterinary Medicine Bahir Dar University Bahir Dar Ethiopia; ^11^ Department of Veterinary Medicine, College of Agricultural Science Woldia University Woldia Ethiopia

**Keywords:** antimicrobial resistance, Ethiopia, methicillin‐resistant *Staphylococcus aureus*, nasopharyngeal carriage, systematic review and meta‐analysis

## Abstract

Methicillin‐resistant *Staphylococcus aureus* (MRSA) nasopharyngeal carriage is a major global health concern linked to severe infections and transmission. However, comprehensive evidence on the burden of MRSA carriage, antimicrobial resistance, and associated risk factors in Ethiopia remains limited. This study aimed to estimate pooled prevalence, resistance pattern, and determinants of nasopharyngeal MRSA carriage. PubMed, ScienceDirect, Scopus, Web of Science, Google Scholar, and gray literature were searched for cross‐sectional studies published between January 2015 and December 2025. Two groups of reviewers screened studies based on predefined criteria. The risk of bias was assessed using the Joanna Briggs Institute tool. Pooled prevalence and resistance proportions were estimated using a random‐effects model, and pooled odds ratios (ORs) were calculated using the Mantel–Haenszel method. Heterogeneity and publication bias were assessed, and a sensitivity analysis was conducted. A total of 1040 records were identified, and 20 studies (6869 participants) were included. The pooled carriage prevalence was 7.3% (95% CI, 5.0–10.8), with substantial heterogeneity (*I* ^2^ = 95.5%). Resistance was highest to tetracycline (55.75%) and lowest to clindamycin (12.66%). Increased odds of carriage were associated with prior hospitalization (OR, 3.49) and antibiotic use (OR, 2.35). Inconsistent variable coding across included studies limited the inclusion of other potential risk factors. Evidence of publication bias was detected, suggesting that the pooled prevalence should be interpreted with appropriate caution. The findings indicate a considerable burden of MRSA and highlight the need for strengthened antimicrobial stewardship, improved surveillance, and targeted prevention efforts in higher‐risk populations. This review was registered in PROSPERO (CRD420251047192).

## Graphical abstract

This systematic review and meta‐analysis show that nasopharyngeal carriage of methicillin‐resistant *Staphylococcus aureus* remains an important public health concern in Ethiopia. High resistance to commonly used antibiotics and significant associations with prior hospitalization and antibiotic use highlight the need for strengthened antimicrobial stewardship, surveillance, and infection prevention.

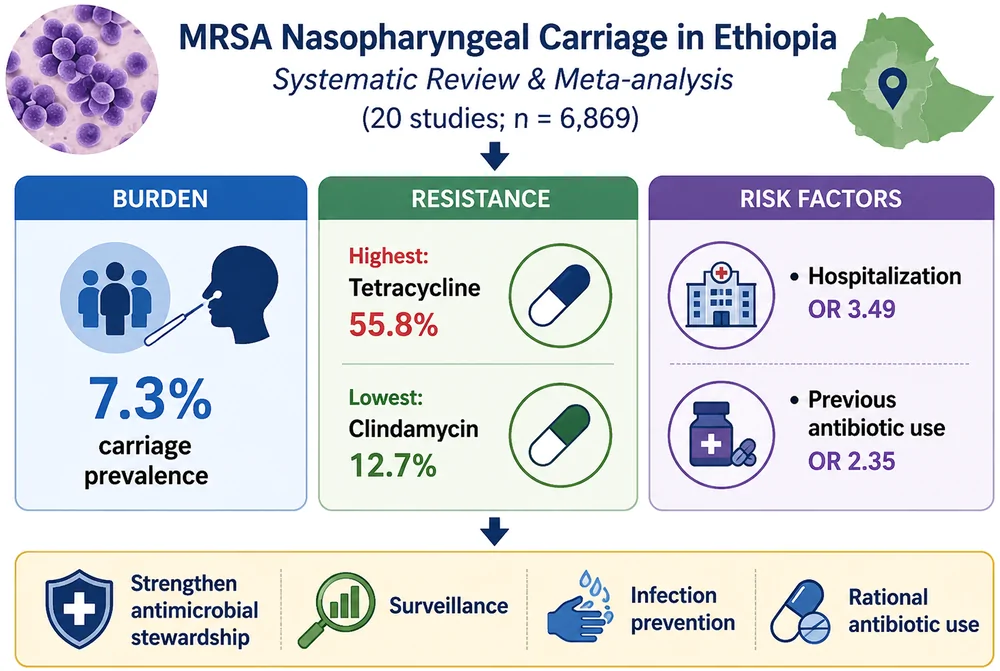

**Abbreviations**
AMR: antimicrobial resistance
CI: confidence interval
MRSA: methicillin‐resistant *Staphylococcus aureus*
OR: odds ratio
PRISMA: preferred reporting items for systematic review and meta‐analysis
SNNPR: southern nations, nationalities, and peoples' region
WHO: World Health Organization

## 1 Introduction

Methicillin‐resistant *Staphylococcus aureus* (MRSA) is a Gram‐positive, facultative anaerobic bacterium that commonly colonizes the skin and nasopharynx of humans (Center for Disease Control [CDC] 2025). It is a major global health threat, responsible for considerable morbidity and mortality. According to the 2019 Global Burden of Disease study, MRSA accounted for approximately 3.5 million disability‐adjusted life years and over 100,000 deaths globally, ranking among the leading causes of antimicrobial resistance (AMR)–related mortality. Together with *Escherichia coli*, *S. aureus* contributed to nearly half of all such deaths in high‐income countries (Murray et al. 2022).

MRSA remains a predominant cause of both hospital‐acquired and community‐associated infections worldwide (David and Daum 2010). Its clinical significance is largely driven by its resistance to nearly all β‐lactam antibiotics, including methicillin, penicillin, and related agents. This resistance is primarily conferred by the *mecA* gene, which encodes an altered penicillin‐binding protein (PBP2a) that has a low affinity for β‐lactams (Turner et al. 2019; Katayama et al. 2000). As a result, treatment options are limited and often require the use of second‐line or more powerful antibiotics, which makes MRSA infections challenging to manage and control in both healthcare and community settings.

The nasopharynx serves as a primary reservoir for MRSA, where asymptomatic colonization plays a critical role in transmission dynamics. Colonized individuals act as potential sources of spread within both healthcare facilities and communities, particularly in crowded environments and through direct contact or contaminated surfaces (Ciftci et al. 2007; Wertheim et al. 2005). Persistent colonization further increases the risk of progression to invasive infections such as pneumonia, sepsis, endocarditis, and skin and soft tissue infections, especially among vulnerable populations, including the elderly, children, and immunocompromised individuals (David and Daum 2010; Kallen 2010).

Despite its epidemiological and clinical importance, the burden of MRSA colonization and its AMR patterns remains insufficiently documented in Ethiopia. Existing evidence is fragmented across different population groups and geographic regions, with reported carriage rates varying considerably between studies. Moreover, prior regional and multicountry syntheses have not comprehensively captured all available Ethiopian primary studies, limiting the representativeness and completeness of the current evidence. Previous meta‐analyses have evaluated antimicrobial susceptibility profiles across a broad range of antibiotics, which are not routinely available or widely used in Ethiopia, such as linezolid, ceftaroline, telavancin, mupirocin, rifampin, and vancomycin (Reta et al. 2019; Azzam et al. 2025). This suggests that resistance patterns reported in broader meta‐analyses may not accurately reflect the local therapeutic landscape of this disease. Consequently, differences in antibiotic availability, prescribing practices, and healthcare access across settings limit the direct applicability of these findings to Ethiopia. These underscore the need for up‐to‐date and context‐specific evidence on MRSA colonization and resistance patterns to commonly used antibiotics to better inform empirical therapy and antimicrobial stewardship. To address these limitations and provide a more context‐specific synthesis, we conducted a systematic review and meta‐analysis focusing on MRSA colonization, resistance patterns for commonly used antibiotics, and associated risk factors in Ethiopia.

Therefore, this study aimed to quantify the pooled prevalence of MRSA nasopharyngeal carriage, analyze its AMR profile against commonly used antibiotics, and identify key factors associated with MRSA colonization in the Ethiopian context. This will provide context‐specific evidence on AMR patterns that may help inform empirical antibiotic selection and antimicrobial stewardship strategies in Ethiopia. The findings will provide crucial evidence for public health authorities, clinicians, and policymakers to design and implement effective prevention, treatment, and antimicrobial stewardship strategies tailored to the Ethiopian context.

## 2 Materials and Methods

### 2.1 Search Strategy

A comprehensive literature search was conducted using bibliographic and citation databases, including Google Scholar, PubMed, Web of Science, Scopus, and ScienceDirect, to identify pertinent articles. The search covered the period from January 1, 2015, to December 18, 2025, to include up‐to‐date data and reflect current MRSA prevalence trends. The comprehensive search strategy used the condition (nasopharyngeal MRSA carriage), context (Ethiopia), and population (humans) (CoCoPop) research question mnemonic frameworks. The complete search strategy and associated keywords are presented in the Supporting Information (Table S1). The search strategy was adapted to align with the specific requirements of each database. An example of the detailed search strategy used for Scopus and PubMed is provided in the Supporting Information (Table S2). This study was reported according to the Preferred Reporting Items for Systematic Reviews and Meta‐Analyses (PRISMA) guidelines (Moher et al. 2009), which is provided in the Supporting Information (Tables S3 and S4). The protocol for this study was registered in PROSPERO (an international prospective register of systematic reviews) on September 30, 2025, with the registration number CRD420251047192.

### 2.2 Inclusion and Exclusion Criteria

All cross‐sectional studies conducted in Ethiopia and published in English, including both peer‐reviewed publications and gray literature sources, were eligible for inclusion in this study. To ensure the incorporation of current and pertinent evidence on the prevalence of MRSA in Ethiopia, only studies conducted after 2015 were considered.

Research articles were excluded for one of the following reasons: (a) reporting the knowledge, attitudes, and practices of MRSA (qualitative studies); (b) articles with insufficient information or records with missing outcomes of interest; (c) personal opinions, correspondence, letters to the editor, proceedings, and reviews; (d) swabs were not taken from the nasopharyngeal area.

### 2.3 Study Selection and Quality Assessment

The quality of the included studies was assessed using the Joanna Briggs Institute's critical appraisal tool, which is designed to evaluate prevalence studies (Munn et al. 2015). The tool has nine items, each measured with four options: “yes,” “no,” “not applicable,” and “unclear,” which assess study quality, including bias, confounding, the validity of exposure and outcome measurements, and the validity of analysis methods. The assessment of the included studies was conducted by two independent teams: Team A (SGE, SKA, DKA, ATZ, BJA, EAT, and EMA) and Team B (YGA, SKA, EAA, HAK, AYA, ATW, and HBA). In this scoring framework, responses categorized as “yes” received a score of 1, whereas responses of “no” or “unclear” (including cases where information was either absent or considered irrelevant) received 0 points. The total scores for each article were aggregated on a scale of 0–9. According to these scores, the articles were classified into three quality categories: good (7–9 points), medium (4–6 points), and low (0–3 points). Disagreements between the two reviewers were resolved through discussion. All studies included in this systematic review and meta‐analysis demonstrated moderate to good methodological quality; therefore, none were excluded for an inadequate study quality (Table S5).

### 2.4 Outcome Variables and Operational Definitions

The primary outcome of this study was the pooled magnitude of nasopharyngeal carriage of MRSA (reported as proportion) in Ethiopia. Secondary outcomes included the pooled antimicrobial susceptibility pattern of MRSA isolates (reported as proportion) and factors associated with nasopharyngeal carriage of MRSA (reported as odds ratio [OR]). In this review, nasopharyngeal carriage was defined as the detection of MRSA from a nasopharyngeal swab specimen regardless of participants' clinical symptom status, unless otherwise specified in the original studies. All studies included in this study used the cefoxitin disc diffusion test as per Clinical and Laboratory Standards Institute (CLSI) guidelines to identify MRSA. A 30‐µg cefoxitin disc was applied to Mueller–Hinton agar inoculated with *S. aureus* isolates. Isolates showing a zone of inhibition greater than 22 mm were interpreted as methicillin‐susceptible, while those with a zone of inhibition of 21 mm or less were classified as methicillin‐resistant. This threshold served as the diagnostic benchmark across all studies included in this review.

### 2.5 Data Extraction

Data were extracted independently by two research teams: Team A (SGE, SKA, DKA, ATZ, BJA, EAT, and EMA) and Team B (YGA, SKA, EAA, HAK, AYA, ATW, and HBA) using a predefined extraction sheet in Microsoft Excel (version 16.54). The extracted data included the name of the first author, year of publication, region where the study was conducted, study subject, sample size, type of transport media, nasopharyngeal carriage rate, and factors associated with the carriage rate, such as history of antibiotic therapy, hospitalization, presence of wound infection, and skin infection. Additionally, the AMR profiles of MRSA isolates were recorded for antibiotics analyzed by at least four authors, including amikacin, chloramphenicol, ciprofloxacin, clindamycin, doxycycline, erythromycin, gentamicin, tetracycline, and trimethoprim‐sulfamethoxazole.

### 2.6 Data Management and Statistical Analysis

Data analysis and visualization were conducted in R software (R Core Team 2024) using *meta* package (Balduzzi et al. 2019). Due to substantial heterogeneity observed across studies, with significant between‐study variability indicated by the inconsistency index (*I* ^2^) of 95.5% (95% CI, 94.1%–96.5%), a random‐effects meta‐analysis model (a model that assumes that the true effect size may vary across studies because of differences in study populations, settings, and methodologies) was used to calculate the overall pooled effect size. Prevalence was analyzed on the natural logarithmic scale (PLN) to stabilize variances and better approximate the normality of the effect‐size distribution. Between‐study variance was estimated using the commonly used estimator called the DerSimonian–Laird approach (DerSimonian and Laird 2015). Given the small number of included studies, Hartung–Knapp–Sidik–Jonkman adjustments were applied to improve confidence interval (CI) coverage and reduce type I error inflation (Doi et al. 2015; Harrer et al. 2021).

The pooled carriage rate of MRSA among individuals was graphically synthesized using a forest plot to display individual study estimates with 95% CIs. Between‐study heterogeneity, attributable to methodological diversity, was quantified using the Cochrane *Q* test (Cochran 1954), and *I* ^2^ statistic (proportion of total variability due to heterogeneity rather than sampling error) (Higgins and Thompson 2002).

Furthermore, subgroup, sensitivity, and meta‐regression analyses were conducted to address the substantial heterogeneity observed in the pooled estimates. Subgroup analysis was conducted by geographic region, study subjects or groups, and type of transport media in the sample to explore sources of variability. Leave‐one‐out sensitivity analysis was used to assess the reliability of the combined results by systematically removing each study in turn. If the CI of the excluded study did not include the overall effect‐size estimate, it was considered to have a significant impact on the results (Mathur and VanderWeele 2020). To further examine the causes of variability among studies, both univariable and multivariable meta‐regression models were used for categories including sample size, publication year, type of transport media, and study region.

Finally, publication bias was assessed using Egger's regression test (Egger et al. 1997) as well as Begg's rank correlation test (Begg and Mazumdar 1994) to check the symmetry of funnel plots. A nonsignificant outcome in Egger's test and Begg's rank correlation test (*p* > 0.05) suggests no evidence of significant publication bias or small‐study effects.

For factor analysis, variables were considered potential factors related to MRSA if at least four studies provided an OR and 95% CI. For studies having data that could be used in the quantitative analysis, Mantel–Haenszel methods (a standard approach for combining ORs from multiple studies to obtain an overall pooled estimate) were used to calculate pooled ORs, and 95% CIs for each factor associated with MRSA colonization (Mantel and Haenszel 1959). Statistical heterogeneity among studies on factors associated with MRSA nasopharyngeal carriage rate was also assessed using the *I* ^2^ statistic. For the AMR analysis, only antibiotics that were tested in at least four independent studies were included in the susceptibility assessment to ensure sufficient data for pooling and improve the precision and stability of the estimated resistance patterns.

## 3 Result

### 3.1 Literature Search and Eligible Studies

**Figure 1:**
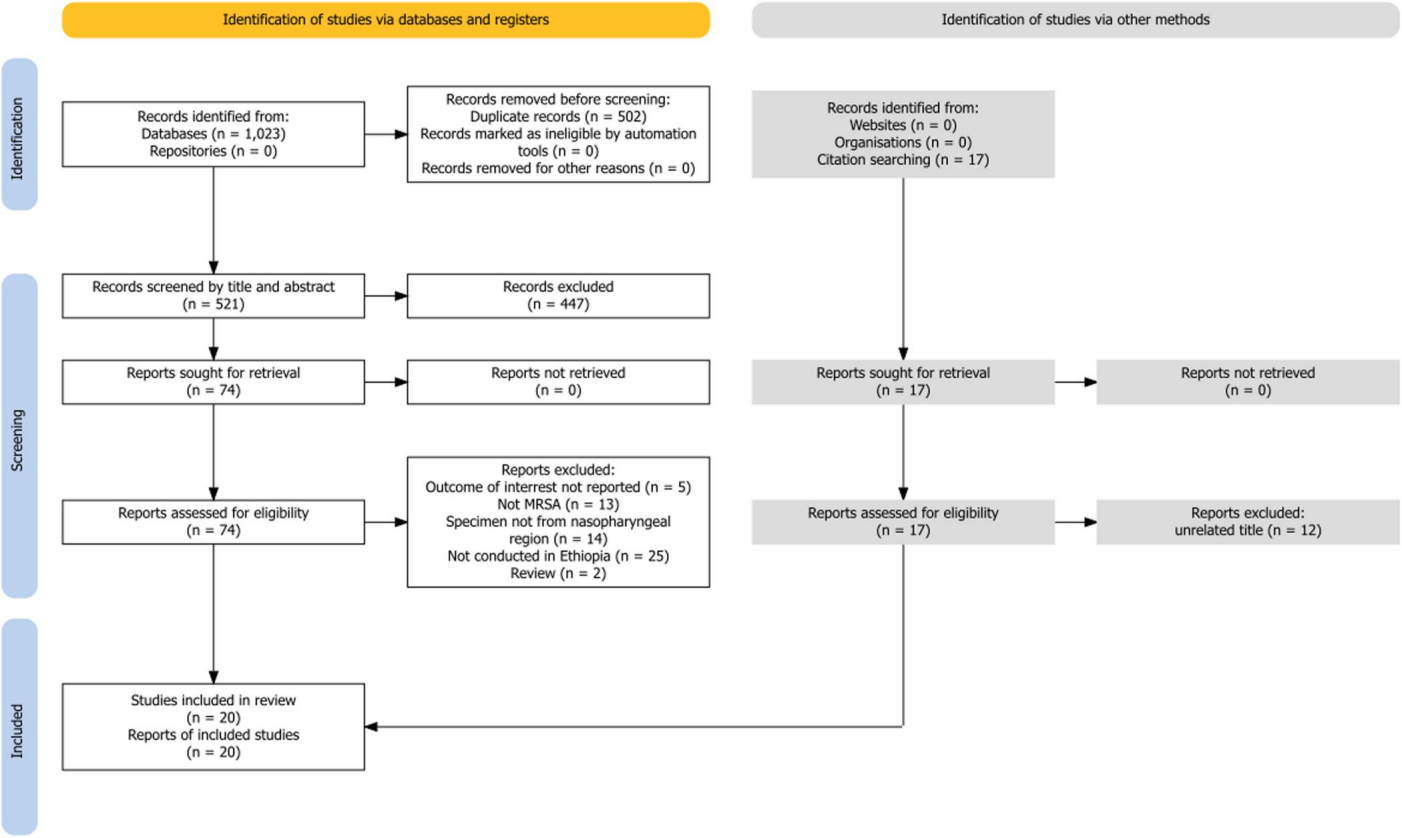
PRISMA flow diagram for study selection (identification, screening, eligibility assessment, and inclusion of studies) in the systematic review and meta‐analysis. MRSA, methicillin‐resistant *Staphylococcus aureus*; PRISMA, preferred reporting items for systematic review and meta‐analysis.

A total of 1040 articles were initially retrieved through electronic searches and manual screening of the reference lists. Approximately 502 articles were excluded because of duplication. After reviewing their titles and abstracts, 447 articles were excluded for unrelated titles or abstracts. The full texts of 74 studies were evaluated for eligibility. Consequently, 59 articles were excluded for the following reasons: they were not conducted in Ethiopia, the outcome of interest was not reported, the swabs were not taken from the nasopharyngeal area, the studies were review articles, or they focused on *S. aureus* rather than MRSA. Five additional studies that met the inclusion criteria were retrieved from other sources (gray literature). Finally, after the methodological quality assessment, 20 studies were included in this systematic review and meta‐analysis (Figure 1).

### 3.2 Characteristics of the Included Studies

A total of 20 studies conducted in Ethiopia were included in this study assessing the nasopharyngeal carriage rate of MRSA, as summarized in Table 1. These studies were published between 2015 and 2025 and were distributed across multiple regions, including Amhara, Oromia, Tigray, and the Southern Nations, Nationalities, and Peoples' Region (SNNPR). Amhara region contributed the highest number of studies (*n* = 9), followed by Oromia (*n* = 6), Tigray (*n* = 3), and SNNPR (*n* = 3). The included studies comprised a combined sample size of 6869 participants, with reported MRSA carriage rates ranging from 1.89% to 37.30%. This wide variation likely reflects substantial between‐study heterogeneity, attributable to differences in the populations studied, including variations in age groups, health status, and study settings. Regarding laboratory procedures, different transport media were utilized, including Amies media, tryptose soya broth, Stuart's media, and direct inoculation methods (Table 1).

**Table 1:** Characteristics of included studies for the nasopharyngeal carriage rate of MRSA in Ethiopia.

Author name	Publication years	Region	Transport media	Sample size	Carriage rate (%)
Abie et al. (2020)	2020	Amhara	Tryptose soya broth	436	4.82
Tigabu et al. (2018)	2018	Amhara	Tryptose soya broth	622	2.25
Adisu et al. (2023)	2023	Oromia	Amies media	351	5.98
Reta et al. (2015)	2015	Amhara	Amies media	300	5.67
Eba et al. (2025)	2025	Oromia	Tryptose soya broth	793	2.27
Efa et al. (2019)	2019	Oromia	Amies media	371	8.36
Gebremedhn et al. (2016)	2016	Tigray	Stuart's media	249	2.41
Gebremeskel et al. (2022)	2022	SNNPR	Direct inoculation	280	9.29
Kahsay et al. (2018)	2018	Tigray	Direct inoculation	384	6.25
Kejela and Dekosa (2022)	2022	Oromia	Direct inoculation	384	18.75
Legese et al. (2018)	2018	Tigray	Direct inoculation	242	5.79
Mamo et al. (2020)	2020	Amhara	Amies media	329	10.64
Manilal et al. (2019)	2019	SNNPR	Direct inoculation	307	20.85
Mekonnen et al. (2025)	2025	Amhara	Amies media	288	11.11
Mekuriya et al. (2022)	2022	SNNPR	Direct inoculation	258	7.36
Muhaba et al. (2022)	2022	Amhara	Amies media	206	28.16
Mulu et al. (2021)	2021	Amhara	Direct inoculation	423	1.89
Shume et al. (2024)	2024	Oromia	Amies media	250	4.80
Weldegebreal et al. (2024)	2024	Oromia	Direct inoculation	270	5.93
Zenebe et al. (2018)	2018	Amhara	Direct inoculation	126	37.30

Abbreviations: MRSA, methicillin‐resistant *Staphylococcus aureus*; SNNPR, southern nations, nationalities, and peoples' region.

### 3.3 Nasopharyngeal Carriage Rate

**Figure 2:**
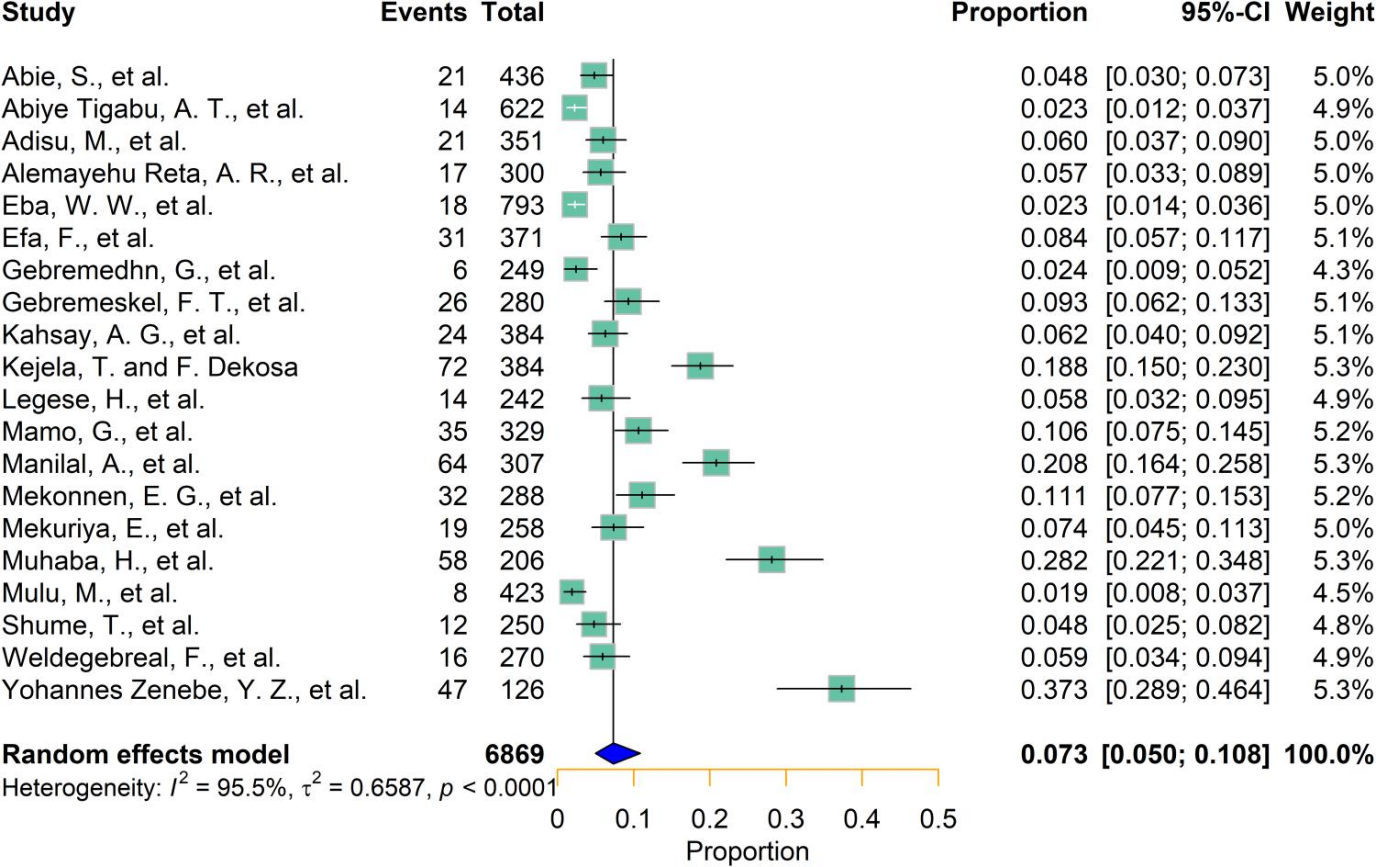
Forest plot for the pooled nasopharyngeal carriage rate of MRSA in humans in Ethiopia. CI, confidence interval; MRSA, methicillin‐resistant *Staphylococcus aureus*.

The apparent prevalence of MRSA carriage ranged from 1.89% (Mulu et al. 2021) to 37.30% (Zenebe et al. 2018) in Ethiopia. The overall pooled nasopharyngeal MRSA carriage rate, as estimated using a random‐effects model, was 7.3% (95% CI, 5.0–10.8) in Ethiopia. Significant heterogeneity was observed with an *I* ^2^ value of 95.5% and a Cochrane *Q* value of 420.84 (*p* < 0.001). On the basis of the findings of this systematic review and meta‐analysis, the predicted prevalence of MRSA in humans is expected to fall within the range of 1.30%–42.0%. This suggests that future studies will likely report results within these intervals (Figure 2). The wider prediction interval likely reflects substantial heterogeneity due to differences in population characteristics, including age, health status, and study settings across the included studies.

### 3.4 Handling Heterogeneity

The meta‐analysis indicated that between‐study variability was high (*Q*, 420.84; *p* < 0.001), indicating statistically significant differences in effect sizes across the included studies. The between‐study variance estimate ($\tau^{2}$) was 0.6587 (95% CI, 0.3756–1.5852), with a substantially high inconsistency index (*I* ^2^ = 95.5%; 95% CI, 94.1%–96.5%).

#### 3.4.1 Subgroup Analysis

To investigate the substantial between‐study heterogeneity, pre‐specified subgroup analyses were conducted using epidemiologically and methodologically relevant moderators, including geographic region (Amhara, Oromia, Tigray, and SNNPR), study population, and specimen transport media. There was notable variation in both the types of media used for specimen transportation to the laboratory and the study populations from which samples were obtained. The highest nasopharyngeal carriage rates were observed in HIV patients (13.8%; 95% CI, 3.5%–54.9%), while the lowest rates were observed in school children (3.1%; 95% CI, 0.8%–11.6%) (Table 2).

**Table 2:** Pooled nasopharyngeal carriage rate of MRSA, stratified by subgroups.

Variables	Included studies	Proportion (95% CI)	Heterogeneity test (*I* ^2^) (%)	*p* value
*Regions*				
Amhara	8	0.0809 [0.0328; 0.1995]	97.0	0.2263
Oromia	6	0.0636 [0.0306; 0.1324]	94.7	
Tigray	3	0.0492 [0.0153; 0.1580]	56.7	
SNNPR	3	0.1146 [0.0291; 0.4509]	92.2	
*Study subjects*				
Janitors	2	0.0554 [0.0106; 0.2886]	0.0	< 0.001
School children	3	0.0308 [0.0082; 0.1158]	79.6	
HIV patients	5	0.1382 [0.0348; 0.5492]	95.7	
College students	4	0.0690 [0.0475; 0.1000]	11.1	
Inpatients	3	0.1270 [0.0506; 0.3185]	85.8	
Health workers	1	0.0579 [0.0348; 0.0962]	—	
Prisoners	1	0.1064 [0.0778; 0.1455]	—	
Pregnant women	1	0.0189 [0.0095; 0.0376]	—	
*Specimen collection media*				
Tryptose soya broth	3	0.0295 [0.0098; 0.0885]	73.5	< 0.001
Amies media	7	0.0911 [0.0521; 0.1592]	93.7	
Stuart's media	1	0.0241 [0.0109; 0.0531]	—	
Direct inoculation	9	0.0942 [0.0481; 0.1844]	95.4	

Abbreviations: CI, confidence interval; MRSA, methicillin‐resistant *Staphylococcus aureus*.

**Figure 3:**
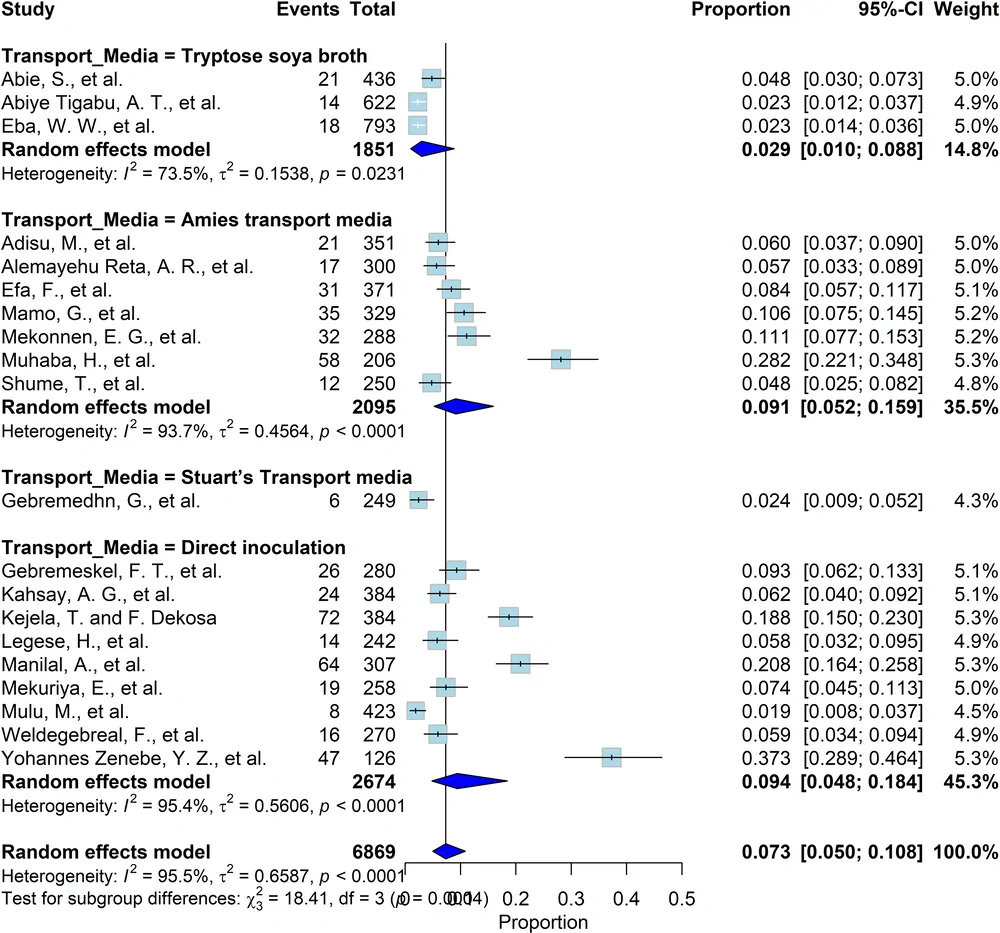
Subgroup analysis stratified by transport media. CI, confidence interval.

The pooled prevalence was highest in studies employing direct inoculation without transport media (9.4%) and those utilizing Amies transport media (9.1%) (Figure 3).

#### 3.4.2 Sensitivity Analysis

**Figure 4:**
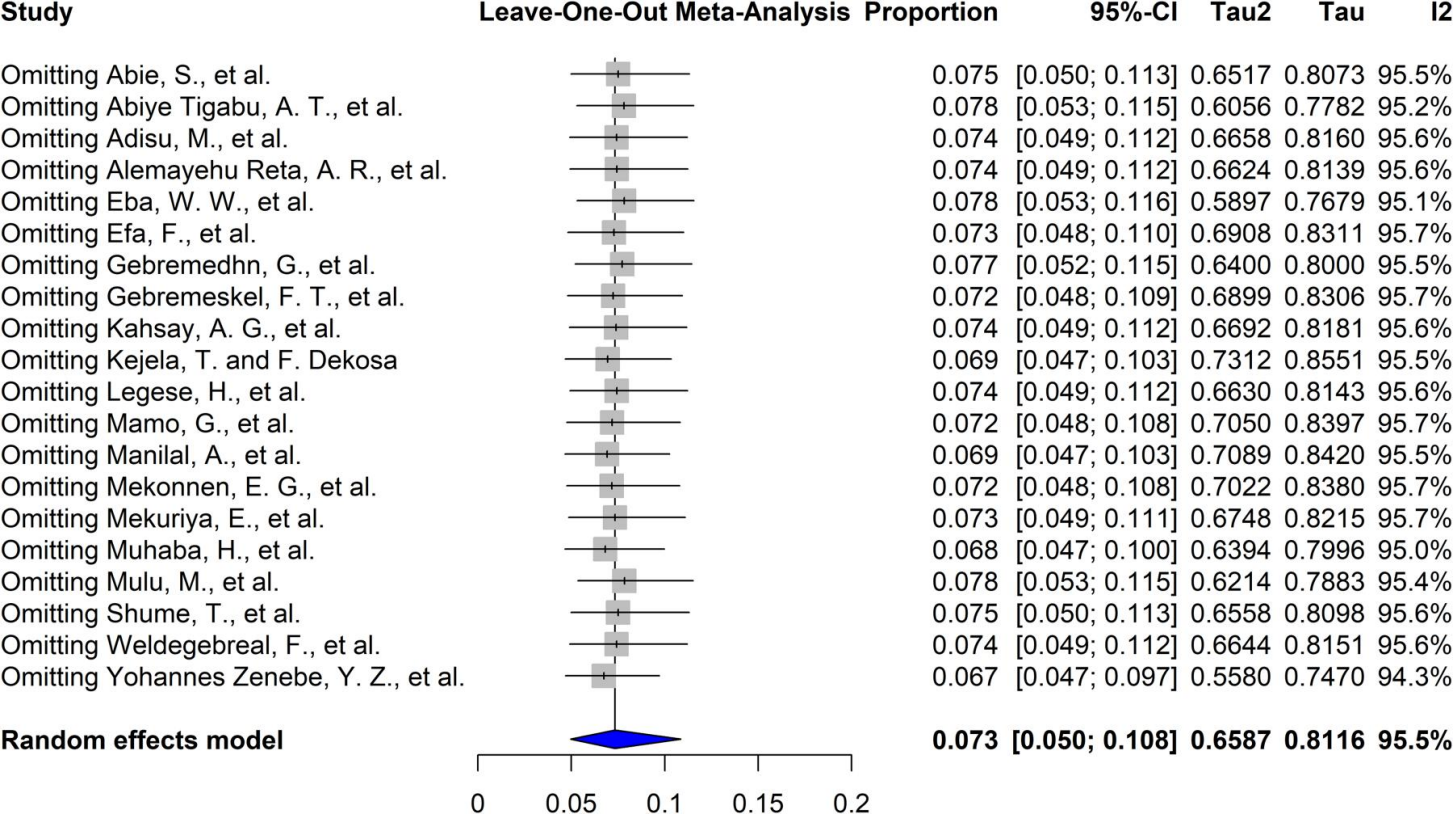
Sensitivity analysis of nasopharyngeal carriage rate of MRSA in humans in Ethiopia. CI, confidence interval; MRSA, methicillin‐resistant *Staphylococcus aureus*.

A leave‐one‐out analysis was performed to assess how each study influenced the overall effect‐size estimate. The meta‐analysis produced a pooled effect size of 0.073 (7.30%), which aligned with the CIs of every individual study included in the analysis. Sensitivity testing further confirmed the stability of these findings, as removing any single study from the analysis did not meaningfully change the overall prevalence estimate (Figure 4). Thus, the meta‐analysis results, including all the studies added in this study, were reliable.

### 3.5 Meta‐Regression Models

To further explore the sources of heterogeneity observed across studies, both uni‐ and multivariable meta‐regression models were performed, as presented in Table 3. In the univariable analysis, both continuous variables (sample size and publication year) and categorical variables (study region, study subjects, and type of specimen transportation media) were examined within a mixed‐effects framework. The univariable regression results indicated that sample size, study subjects, and transport media were associated with between‐study variability (*p* < 0.25). Among these, study subjects emerged as the most influential moderator, explaining 58.25% of the observed heterogeneity (*R* ^2^ = 58.25%). Sample size also contributed substantially, accounting for 45.36% of the observed variability. Transport media explained 24.96% of the heterogeneity, suggesting that differences in specimen‐handling procedures may influence reported prevalence estimates. In contrast, publication year and study region variability did not demonstrate meaningful contributions to the observed heterogeneity.

**Table 3:** Univariable and multivariable meta‐regression analysis results of nasopharyngeal carriage rate of MRSA in humans in Ethiopia.

Moderators	Category	Univariable regression		Multivariable regression	
		*R* ^2^ (%)	Coefficient (95% CI)	*R* ^2^ (%)	Coefficient (95% CI)
Region	Amhara	0.00	Reference		
	Oromia		−0.25 (−1.23; 0.73)		
	SNNPR		0.33 (−0.89; 1.55)		
	Tigray		−0.58 (−1.83; 0.67)		
Sample size		45.36	−0.003 (−0.006; −0.001)**		−0.002 (−0.006; 0.001)
Publication year		0.00	0.006 (−0.14; 0.15)		
Study subjects	College students	58.25	Reference		Reference
	Janitors		−0.17 (−1.49; 1.14)		0.09 (−1.02; 1.19)
	HIV patients		0.80 (−0.23; 1.82)		0.99 (0.19; 71.79)**
	School children		−0.75 (−1.93; 0.43)		−0.02 (−1.20; 1.16)
	Inpatients		0.65 (−0.49; 1.80)		0.68 (−0.19; 1.54)
	Health workers		−0.12 (−1.87; 1.62)		−0.34 (−1.64; 0.96)
	Prisoners		0.49 (−1.17; 2.14)		0.72 (−0.58; 2.02)
	Pregnant women		−1.24 (−3.10; 0.61)		−1.07 (−2.44; 0.31)
Transport media	Amies media	24.96	Reference		Reference
	Direct inoculation		0.04 (−0.74; 0.82)		0.26 (−0.39; 0.92)
	Stuart's media		−1.33 (−3.15; 0.49)		−1.93 (−3.22; −0.64)**
	Tryptose soya broth		−1.14 (−2.22; −0.05)		0.03 (−1.39; 1.45)
Full model			Transport media + Sample size + Study subjects	85.84	

Abbreviations: CI, confidence interval; MRSA, methicillin‐resistant Staphylococcus aureus; SNNPR, southern nations, nationalities, and peoples' region.

** Significant variables or categories; *R* ^2^, coefficient of determination.

In the multivariable meta‐regression model, which included sample size, study subjects, and transport media, a substantial proportion of heterogeneity was explained (*R* ^2^ = 85.84%). After adjustment for covariates, studies conducted among people living with HIV showed a significantly higher MRSA carriage rate compared to the reference group (college students) (*β* = 0.99; 95% CI, 0.19–1.79). Additionally, the use of Stuart's transport media was associated with significantly lower reported carriage rates compared to Amies media (*β* = −1.93; 95% CI, −3.22 to −0.64) (Table 3).

### 3.6 Publication Bias

**Figure 5:**
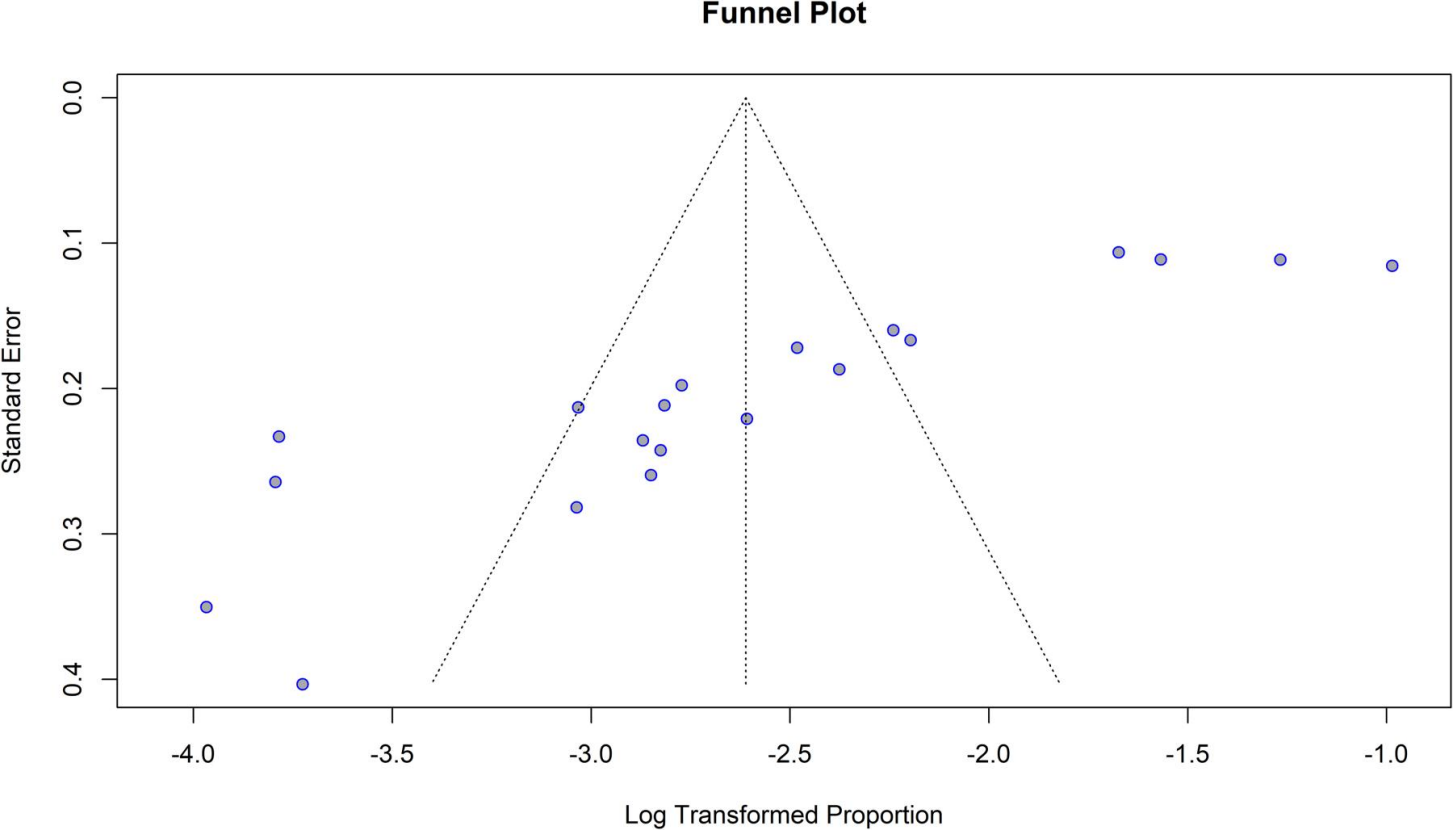
Funnel plot of the pooled nasopharyngeal carriage rate of methicillin‐resistant *Staphylococcus aureus* in humans in Ethiopia.

Visual inspection of the funnel plots revealed an asymmetrical distribution of effect‐size estimates across the included studies (Figure 5). To complement the subjective assessment, quantitative statistical tests were performed using Egger's regression analysis, which produced a value of (*t* = −9.10; *p* < 0.001) and Begg's rank correlation test (*z* = −3.63; *p* = 0.0003). Both analyses indicated a statistically significant asymmetry in the aggregated data, providing strong evidence of publication bias or small‐study effects that may have influenced the meta‐analysis results.

**Figure 6:**
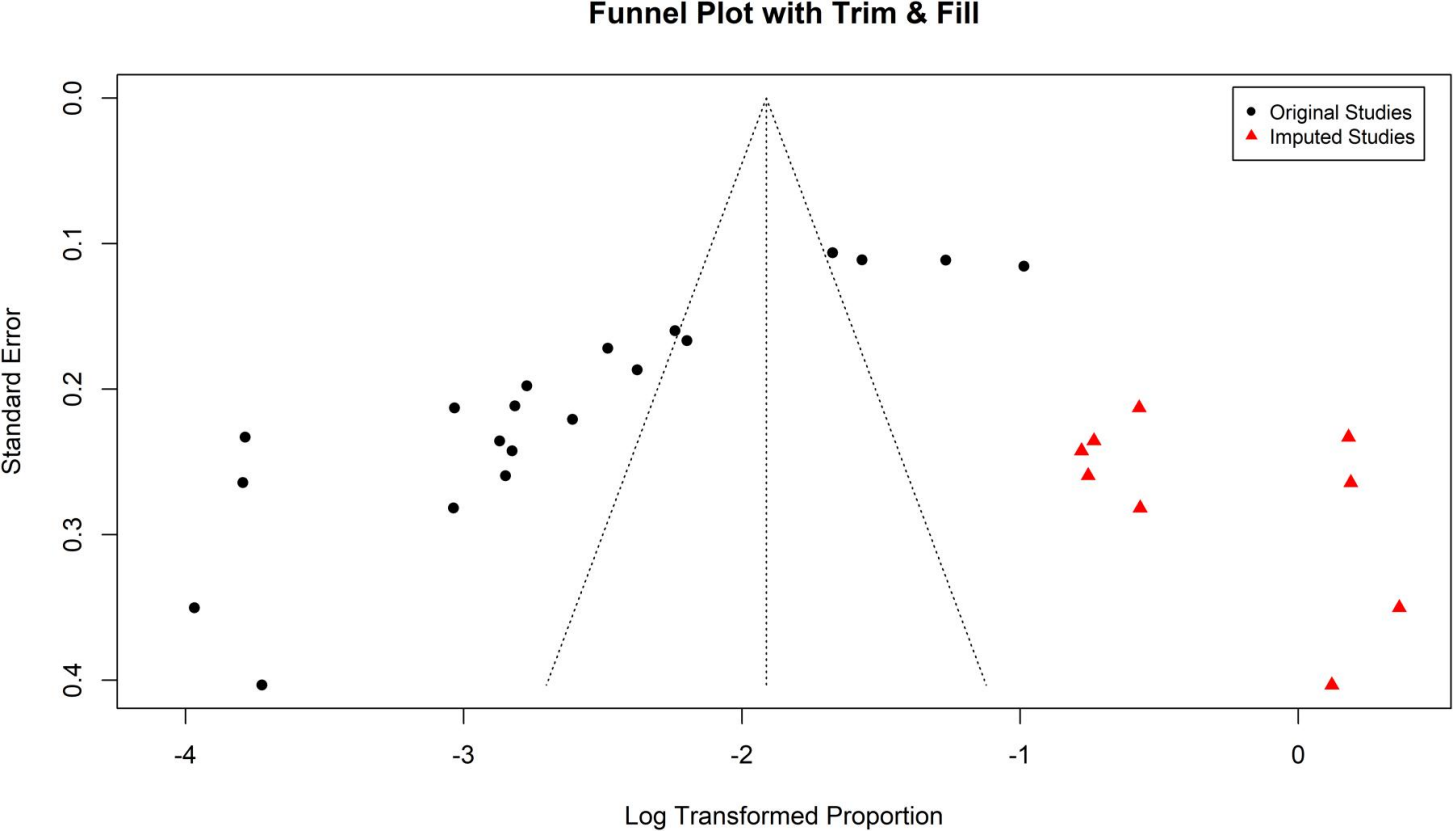
Trim‐and‐fill analysis by imputing imaginary studies.

To address the issue of publication bias, a trim‐and‐fill analysis was conducted, which resulted in the imputation of nine imaginary studies on the right side of the funnel plot (Figure 6). Ultimately, the overall pooled prevalence of MRSA in Ethiopia, based on all 29 studies (20 observed and 9 imputed), was found to be 14.77% (95% CI, 8.96%–24.37%).

### 3.7 AMR Profile

All studies included in this study employed culture‐based methods for the identification of MRSA isolates, with antimicrobial susceptibility testing performed using the Kirby–Bauer disc diffusion technique. A total of 3760 MRSA isolates were analyzed to determine resistance patterns against commonly prescribed antibiotics in Ethiopia.

The included studies assessed resistance to a range of antimicrobial agents, including amikacin, chloramphenicol, ciprofloxacin, clindamycin, doxycycline, erythromycin, gentamicin, tetracycline, and trimethoprim‐sulfamethoxazole. Among these, erythromycin (*n* = 17 studies) and trimethoprim‐sulfamethoxazole (*n* = 15 studies) were the most frequently evaluated antibiotics, followed by tetracycline and chloramphenicol (*n* = 12 studies each). The number of MRSA isolates tested varied across antimicrobial classes (Table 4).

**Table 4:** Pooled antimicrobial resistance rate of MRSA isolated from the nasopharyngeal area in humans in Ethiopia.

Type of antimicrobial	Group of antimicrobial agents	No. of studies	No. of MRSA isolates tested	Resistance rate	
				Pooled prevalence (%) (95% CI)	*I* ^2^ (%) (*p* value)
Amikacin	Aminoglycoside	4	163	15.30 (2.79; 83.94)	86.3 (< 0.01)
Chloramphenicol	Chloramphenicol	12	305	15.56 (8.14; 29.75)	72.6 (< 0.01)
Ciprofloxacin	Fluoroquinolones	13	304	28.02 (17.33; 45.30)	81.1 (< 0.01)
Clindamycin	Lincosamide	14	359	12.66 (8.21; 19.53)	27.2 (0.16)
Doxycycline	Tetracyclines	6	217	36.18 (17.97; 72.87)	83.1 (< 0.01)
Erythromycin	Macrolides	17	473	44.42 (33.24; 59.35)	90.1 (0.01)
Gentamicin	Aminoglycosides	13	367	22.89 (14.97; 33.01)	60.0 (0.0028)
Tetracycline	Tetracyclines	12	360	55.75 (45.54; 68.25)	80.9 (< 0.01)
Trimethoprim‐sulfamethoxazole	Folic acid metabolism inhibitors	15	431	49.88 (37.82; 65.80)	83.2 (< 0.01)

Abbreviations: CI, confidence interval; MRSA, methicillin‐resistant *Staphylococcus aureus*.

The pooled result showed considerable variability in resistance patterns across antimicrobial agents. The highest level of resistance was observed for tetracycline (55.75%; 95% CI, 45.54–68.25), followed by trimethoprim‐sulfamethoxazole (49.88%; 95% CI, 37.82–65.80) and erythromycin (44.42%; 95% CI, 33.24–59.35). Moderate resistance levels were identified for doxycycline, ciprofloxacin, and gentamicin, whereas relatively lower resistance was observed for chloramphenicol and amikacin. Clindamycin showed the lowest pooled resistance (12.66%; 95% CI, 8.21–19.53), indicating comparatively better susceptibility among MRSA isolates.

Substantial heterogeneity was observed across most pooled estimates, with *I* ^2^ values ranging from 60% to 90.10%, except for clindamycin, which demonstrated low to moderate heterogeneity (*I* ^2^ = 27.20%). This variability may be attributed to differences in study populations, geographic settings, antimicrobial usage patterns, and laboratory methodologies across the included studies (Table 4).

### 3.8 Risk Factors Associated With the Nasopharyngeal Carriage Rate of MRSA

Several included studies evaluated potential risk factors associated with MRSA nasopharyngeal colonization in Ethiopia, as summarized in Table 5. Fourteen studies assessed the effect of prior hospitalization, while 11 studies investigated previous antibiotic therapy.

**Table 5:** Factors associated with the nasopharyngeal carriage rate of MRSA in humans in Ethiopia.

Risk factor	Category	No. of studies	Pooled OR (95% CI)	*I* ^2^ (%) (*p* value)
History of hospitalization	No	14	Reference	59.8 (0.0022)
	Yes		4.48 (2.62–7.67)**	
Previous antibiotic therapy	No	11	Reference	70.5 (0.0002)
	Yes		2.41 (1.22–4.73)**	
Presence of a wound	No	5	Reference	81.1 (0.0003)
	Yes		1.95 (0.40–9.51)	
Presence of respiratory infection	No	6	Reference	85.0 (< 0.0001)
	Yes		3.62 (0.56–23.59)	
Sex	Female	13	Reference	13.3 (0.3110)
	Male		1.02 (0.76–1.37)	
Presence of skin lesions	No	4	Reference	88.0 (< 0.0001)
	Yes		2.76 (0.25–30.68)	

Abbreviations: CI, confidence interval; MRSA, methicillin‐resistant Staphylococcus aureus; OR, odds ratio.

**Significant at 95% level of significance.

The pooled random‐effects meta‐analysis demonstrated that prior hospitalization and previous antibiotic use were significantly associated with increased odds of MRSA nasopharyngeal carriage. Individuals with a history of hospitalization had more than fourfold higher odds of MRSA colonization (OR, 4.48; 95% CI, 2.62–7.67), whereas those with prior antibiotic exposure had more than twofold higher odds of carriage (OR, 2.41; 95% CI, 1.22–4.73). In contrast, the presence of wounds (OR, 1.95; 95% CI, 0.40–9.51), respiratory infection (OR, 3.62; 95% CI, 0.56–23.59), skin lesions (OR, 2.76; 95% CI, 0.25–30.68), and male sex (OR, 1.02; 95% CI, 0.76–1.37) were not significantly associated with MRSA nasopharyngeal carriage (Table 5).

Moderate to substantial between‐study heterogeneity was observed for most risk factor analyses (*I* ^2^ ranging from 59.8% to 88.0%), whereas low heterogeneity was observed for sex (*I* ^2^ = 13.3%). This variability likely reflects differences in study populations, clinical settings, and methodological characteristics among the included studies.

**Figure 7:**
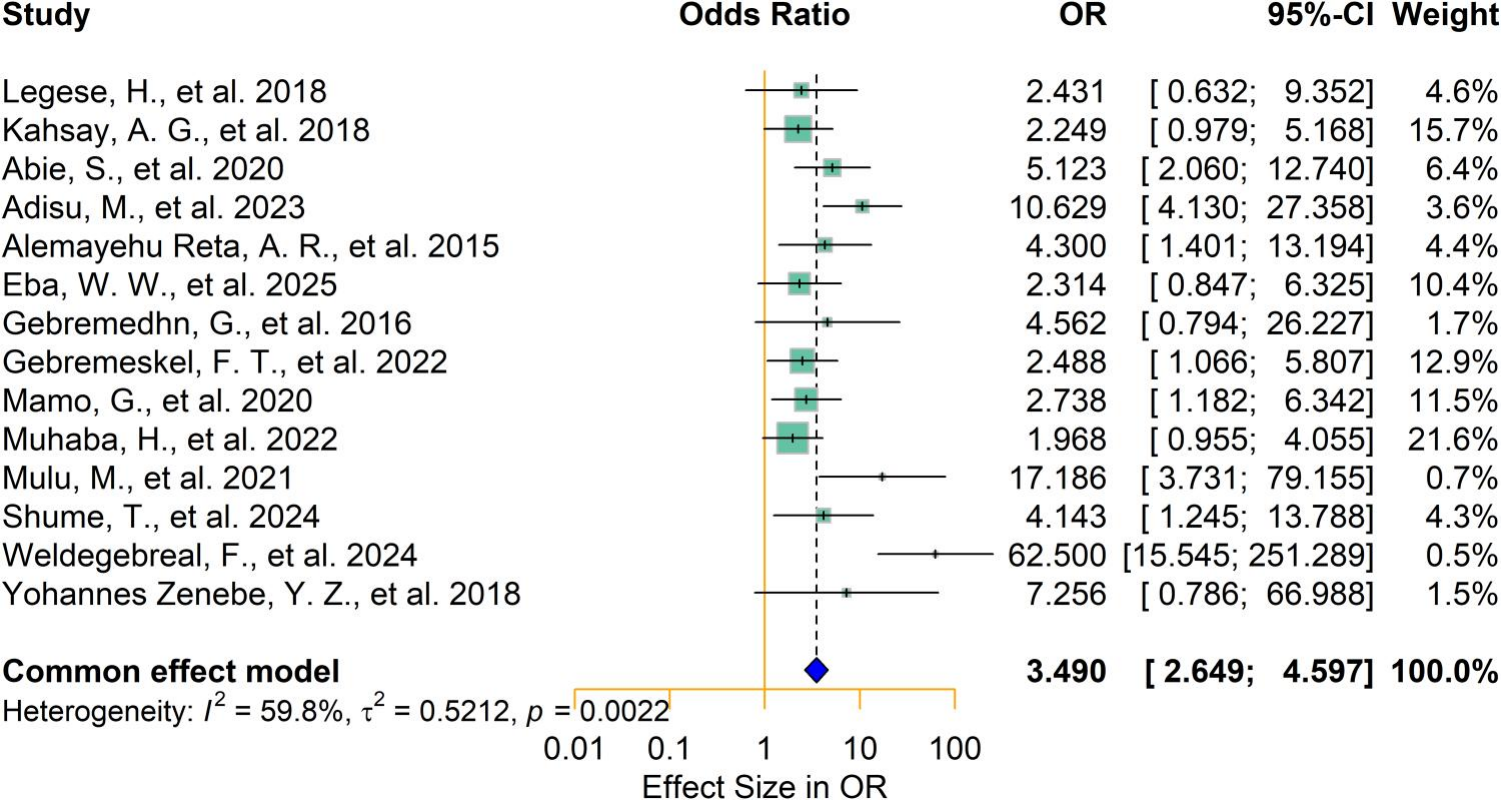
Association of previous hospitalization with the risk of MRSA colonization in humans in Ethiopia. CI, confidence interval; MRSA, methicillin‐resistant *Staphylococcus aureus*; OR, odds ratio.

The pooled random‐effects meta‐analysis showed that previous hospitalization was significantly associated with MRSA nasopharyngeal carriage. Individuals with a history of hospitalization had approximately 4.5‐fold higher odds of MRSA colonization than those without a history of hospitalization (OR, 4.48; 95% CI, 2.62–7.67). Moderate between‐study heterogeneity was observed (*I* ^2^ = 59.8%; *p* = 0.0022) (Figure 7 and Table 5).

**Figure 8:**
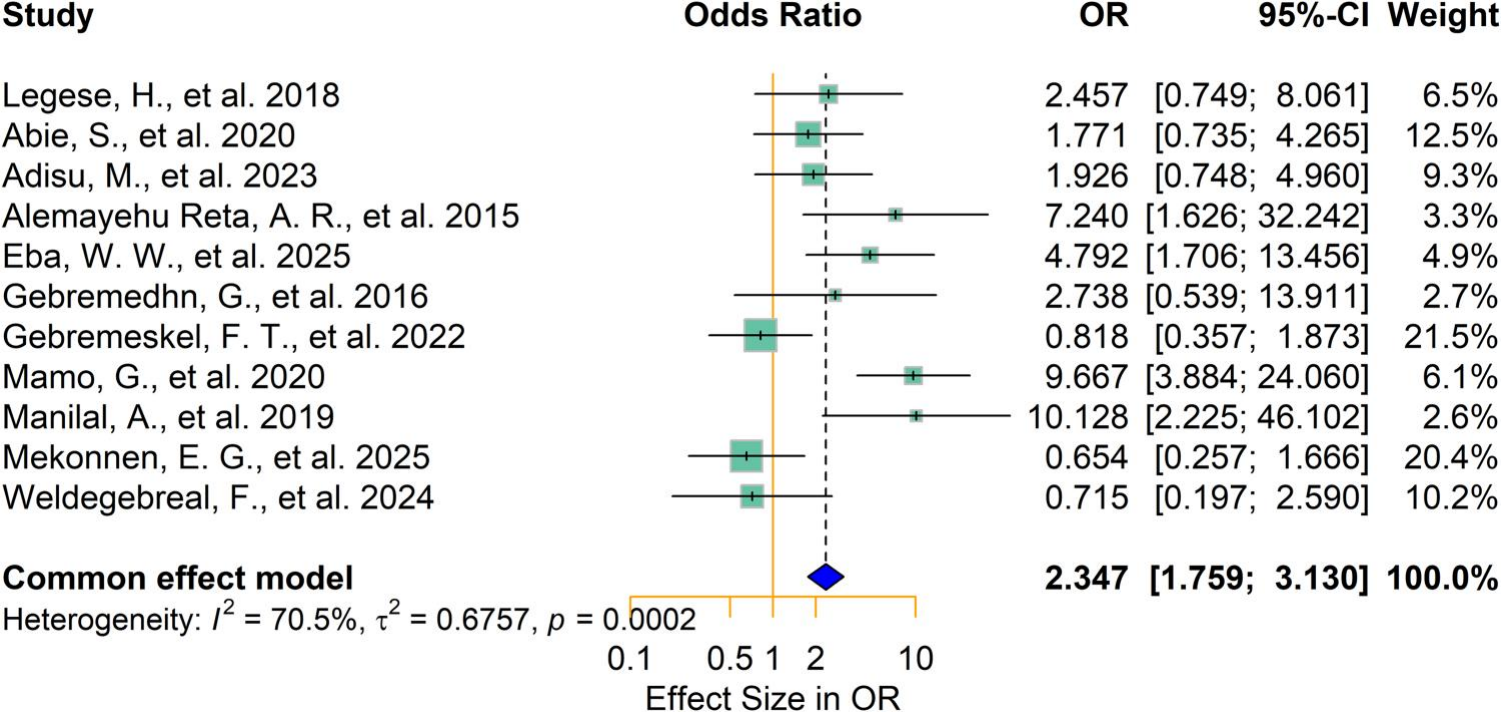
Association of previous antibiotic therapy with the risk of MRSA nasopharyngeal colonization in humans in Ethiopia. CI, confidence interval; MRSA, methicillin‐resistant *Staphylococcus aureus*; OR, odds ratio.

The pooled random‐effects meta‐analysis indicated that previous antibiotic therapy was significantly associated with an increased risk of MRSA nasopharyngeal carriage. Individuals with a history of antibiotic use had approximately 2.4‐fold higher odds of MRSA colonization than those without prior antibiotic exposure (OR, 2.41; 95% CI, 1.22–4.73). Moderate between‐study heterogeneity was observed among the included studies (*I* ^2^ = 70.5%; *p* = 0.0002) (Figure 8 and Table 5).

### 3.9 Trends of MRSA Prevalence Over Time

**Figure 9:**
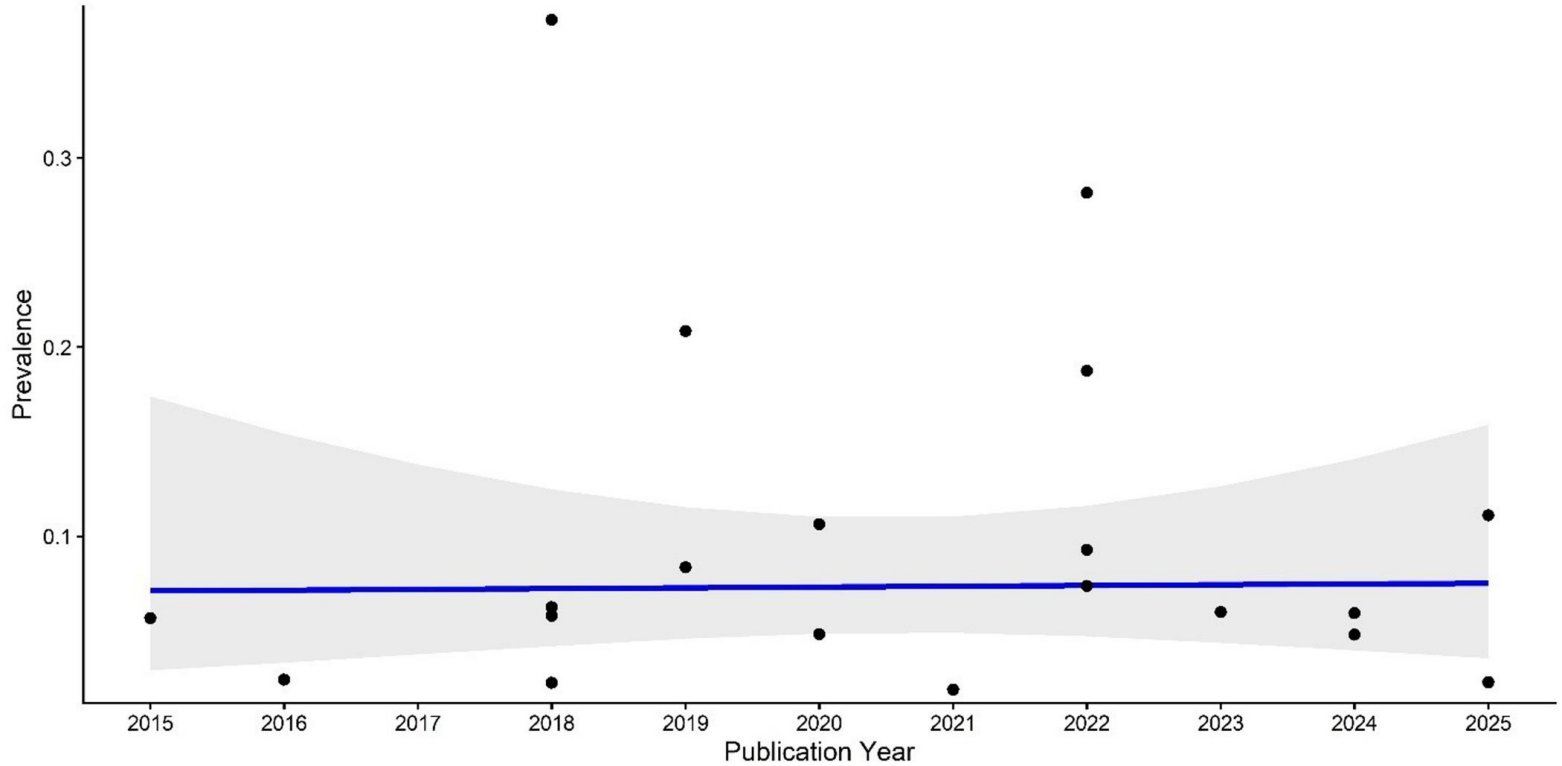
Trends in methicillin‐resistant *Staphylococcus aureus* prevalence predicted based on existing studies over time (2015–2025).

The meta‐regression analysis showed no significant temporal trend (*p* = 0.9369) in prevalence across the study period (2015–2025). The predicted prevalence remained relatively constant over time, with a nearly horizontal regression slope, suggesting no evidence of increasing or decreasing prevalence. However, substantial between‐study variability was observed, indicating that factors other than publication year likely explain the heterogeneity in reported prevalence estimates (Figure 9).

## 4 Discussion

MRSA remains an important public health concern due to its asymptomatic nasopharyngeal colonization and resistance to commonly used antibiotics. This systematic review and meta‐analysis assessed the prevalence of nasopharyngeal MRSA carriage, AMR patterns, and associated risk factors in Ethiopia, incorporating evidence from 20 studies with a total of 6869 participants. The findings indicate variability in colonization rates across population groups and demonstrate notable resistance to several commonly used antibiotics. In addition, factors such as prior antibiotic use, recent hospitalization, respiratory infection, and presence of wounds were consistently associated with an increased likelihood of MRSA colonization. Taken together, these results provide an overall picture of MRSA epidemiology in Ethiopia and suggest the need for strengthened surveillance, improved antimicrobial stewardship, and targeted infection prevention efforts, particularly in higher‐risk populations.

The overall nasopharyngeal MRSA carriage rate was 7.34% (95% CI, 4.97–10.85) in Ethiopia. The findings of this study were consistent with previous evidence reported from Tanzania (8.5%) (Joachim et al. 2017) but were lower than the 42% prevalence in Iran (Akbari and Mohammadi 2023), 39.0% in India (S. Li et al. 2014), and 35.6% in Saudi Arabia (Yousef et al. 2013). This could be attributed to variations in antibiotic prescribing practices, infection prevention strategies, hygiene standards, and diagnostic capabilities (David and Daum 2010; Gesualdo et al. 2013). These differences may also reflect variations in healthcare infrastructure, antimicrobial stewardship programs, study populations, and local epidemiological settings, all of which can influence MRSA transmission dynamics and the observed prevalence across countries. The findings of this study showed that the MRSA nasopharyngeal carriage rate was significant, highlighting the need for immediate interventions, such as strengthening antimicrobial stewardship programs, undertaking national antimicrobial surveillance, and implementing One Health initiatives.

The publication bias assessment indicated significant asymmetry in the funnel plot, supported by both Egger's and Begg's tests. Furthermore, the trim‐and‐fill analysis increased the pooled prevalence estimate from 7.34% to 14.77%, suggesting that publication bias or small‐study effects may have led to an underestimation of the true MRSA carriage rate. However, because the trim‐and‐fill procedure provides an exploratory adjusted estimate based on statistical assumptions, the corrected estimate should be interpreted with caution.

The prevalence of MRSA carriage varies considerably across different population groups in Ethiopia. The highest nasopharyngeal carriage rates were observed in HIV patients (13.80%; 95% CI, 3.50%–54.9%), compared to the lowest rates in studies among school children (3.10%; 95% CI, 0.80%–11.6%). In line with this finding, evidence suggests that HIV‐positive individuals may have a 6–18‐fold increased susceptibility to MRSA compared to the general population (Crum‐Cianflone et al. 2007; Sabbagh et al. 2019). This high risk may be attributed to differences in host immunity, healthcare contact, and hygiene practices (Sissolak et al. 2002). Additionally, studies conducted in Ethiopia have shown that MRSA nasopharyngeal colonization is prevalent, particularly among hospitalized patients and individuals with frequent healthcare contact (Gebremedhn et al. 2016; Mekonnen et al. 2025).

In this study, the highest rate of antibiotic resistance among MRSA isolates was observed against tetracycline, whereas clindamycin exhibited relatively low resistance. The relatively low resistance to clindamycin may reflect its limited availability and use compared with other commonly prescribed antibiotics. In contrast, the high level of tetracycline resistance may be partly attributed to its widespread use in Ethiopia. Previous evidence indicates that less frequently prescribed antibiotics, such as linezolid, tend to confer higher susceptibility, whereas commonly accessible drugs show higher resistance due to increased exposure (Brown et al. 2021; Falagas et al. 2013). Tetracycline is commonly utilized because of its low cost, broad‐spectrum activity, and wide availability in both healthcare facilities and informal drug outlets (Gebeyehu et al. 2015; Ayalew 2017). In addition, Tetracycline is extensively used in livestock production, which may further promote the emergence and spread of resistant strains (Z. Li et al. 2018; Schwarz et al. 2001). These findings are consistent with reports from other East African settings, such as Kenya, which reported reduced susceptibility of MRSA isolates to commonly used antibiotics, including tetracycline (Wangai et al. 2019).

In addition, trimethoprim‐sulfamethoxazole and erythromycin showed moderate resistance against MRSA isolates. The erythromycin resistance of MRSA isolates was consistent with previous evidence from Africa (Wangai et al. 2019; Ezeh et al. 2023). These findings highlight the importance of using antimicrobials only when proven effective, considering alternative therapies where appropriate, and enforcing tighter controls on pharmacies and drug vendors to reduce nonprescription antibiotic use and encourage proper prescribing practices.

This study also identified key risk factors significantly associated with nasopharyngeal MRSA colonization in Ethiopia. Prior hospitalization, previous antibiotic therapy, skin lesions, history of respiratory infection, and wounds were significantly associated with an increased risk of MRSA infection. These findings are consistent with evidence from other settings (Fukuta et al. 2012; Hu et al. 2022; Zervou et al. 2014). For instance, a systematic review and meta‐analysis incorporating 29 studies with 76,913 patients in the United States found that prior hospitalization (OR, 2.40; 95% CI, 1.30–4.70) (McKinnell et al. 2013). Additionally, a systematic review and meta‐analysis study performed in China (Hu et al. 2022) reported a significant association between prior antibiotic use and MRSA nasopharyngeal colonization (OR, 2.69; 95% CI, 2.09–3.45). The observed associations suggest that individuals with recent healthcare contact or prior antibiotic exposure are important target groups for infection prevention and control strategies. Strengthening screening, hygiene practices, and rational antibiotic use in these high‐risk populations may help reduce MRSA transmission. These findings also highlight the importance of antimicrobial stewardship efforts, which help minimize unnecessary antibiotic exposure and limit the development and spread of resistance.

This study has limitations that should be considered when interpreting the findings. Although the multivariable meta‐regression explained a substantial proportion of the between‐study heterogeneity (*R* ^2^ = 85.84%), some residual variability remained. Therefore, the pooled prevalence should be interpreted with caution, as differences in study populations and methodological characteristics may have influenced the reported estimates. In addition, the literature search was restricted to English‐language publications, which may introduce language bias, although this is likely minimal, given that most academic research in the Ethiopian context is published in English. Although publication bias was detected, and the trim‐and‐fill analysis suggested that the pooled prevalence may have been underestimated, the adjusted estimate should be interpreted with caution because the trim‐and‐fill method provides an exploratory statistical correction based on underlying assumptions. The geographic distribution of the included studies was also limited to a few regions, which may reduce the national representativeness of the findings for Ethiopia. The analysis of risk factors was further constrained by inconsistencies in variable definitions and categorizations across studies, which is difficult to pool important determinants, such as comorbidities, socioeconomic status, healthcare worker contact, and hygiene practices. Furthermore, for some antibiotics, resistance estimates were based on a limited number of studies, resulting in wide CIs and reduced precision of the pooled results.

## 5 Conclusion

This systematic review and meta‐analysis indicate that MRSA nasopharyngeal carriage remains an important public health concern in Ethiopia, emphasizing the need to strengthen antimicrobial stewardship, enhance surveillance systems, and implement targeted infection prevention and control measures, particularly in high‐risk populations. Although the findings should be interpreted with caution because of the observational nature of the included studies and substantial between‐study heterogeneity, they provide valuable context‐specific evidence to inform public health planning and guide future research. Further well‐designed prospective studies incorporating molecular characterization of MRSA strains, including SCCmec typing, *spa* typing, and detection of virulence factors such as Panton–Valentine leukocidin, are warranted to improve understanding of MRSA epidemiology and support evidence‐based prevention and control strategies in Ethiopia.

## Author Contributions

**Simachew Getaneh Endalamew:** conceptualization, writing – original draft, methodology, validation, visualization, writing – review and editing, software, formal analysis, data curation. **Sofiya Ayalew Kebede:** methodology, software, validation, visualization, writing – review and editing, data curation, supervision. **Solomon Keflie Assefa:** writing – review and editing, methodology, validation, visualization, software, supervision, data curation. **Adem Tsegaw Zegeye:** visualization, writing – review and editing, validation, supervision, data curation. **Belayneh Jejaw Abate:** writing – review and editing, visualization, validation, supervision, data curation. **Dejen Kahsay Asgedom:** writing – review and editing, visualization, validation, supervision, data curation. **Eliyas Addisu Taye:** writing – review and editing, visualization, validation, supervision, data curation. **Endalew Minwuye Andargie:** writing – review and editing, visualization, validation, data curation, supervision. **Eyob Akalewold Alemu:** writing – review and editing, visualization, validation, data curation, supervision. **Halima Ayalew Kebede:** conceptualization, visualization, writing – review and editing, validation, supervision. **Alebachew Tilahun Wassie:** writing – review and editing, visualization, validation, data curation, supervision. **Andnet Yirga Assefa:** writing – review and editing, visualization, validation, data curation, supervision. **Yihenew Getahun Ambaw:** writing – review and editing, visualization, validation, data curation, supervision. **Helen Brhan Alemaw:** writing – review and editing, visualization, validation, methodology, conceptualization, data curation, supervision, software, writing – original draft.

## Funding

The authors have nothing to report.

## Ethics Statement

The authors have nothing to report.

## Consent

We used published articles for further analysis. The participants of this study were articles. Consequently, consent was not obtained from the study participants.

## Conflicts of Interest

None declared.

## Declaration of Generative AI and AI‐Assisted Technologies

During the preparation of this work, the author(s) used ChatGPT in order to assist with grammatical editing and to improve the clarity and coherence of the text. After using this tool/service, the author(s) reviewed and edited the content as needed and take(s) full responsibility for the content of the published article.

## Supporting information


Supporting File

## Data Availability

The data that support the findings of this study are available in the Supporting Information of this article.
