# Supplementary material for: Nasopharyngeal Carriage Rate, Risk Factors, and Co‐Resistance Patterns of Methicillin‐Resistant Staphylococcus aureus in Ethiopia: Systematic Review and Meta‐Analysis
Source: Microbiologyopen. 2026 Sep 9;15(5):e70409. doi: 10.1002/mbo3.70409 (PMC13555809; doi:10.1002/mbo3.70409)
Supplement: Supplementary file 1 [file MBO3-15-e70409-s001.docx]

### **Research Question:** What is the **nasopharyngeal carriage rate**, **associated risk factors**, and co-resistance patterns of **methicillin-resistant** Staphylococcus aureus **(MRSA) in Humans** in Ethiopia?

### **Table S1: Detailed Search Strategy with Mnemonics (CoCoPop Framework)**

| Mnemonics | CoCoPop (**condition**, **context/setting**, **population**) | | |
| --- | --- | --- | --- |
|  | **Condition** | **Population** | **Context / Setting** |
| **Search Terms** | | | |
| **MRSA Keywords** | MRSA; Methicillin-resistant Staphylococcus aureus | Human*; Patient*; Child*; Adult* | Ethiopia |
| **Synonyms** | Methicillin resistant Staphylococcus aureus; Methicillin resistant S. aureus; Methicillin-resistant S. aureus; MRSA infection; Antibiotic-resistant S. aureus; Drug-resistant Staphylococcus aureus; MRSA Disease*; MRSA Infection* | Individual*; Neonates; Pediatric; Healthcare workers; Community members | Ethiopian Hospitals; Ethiopian Health facilities; Ethiopia Community setting |
| **Nasopharyngeal carriage rate-Related Terms** | Nasal carriage; Nasopharyngeal coloniz*; Nasopharyngeal colonis*; Nasopharyngeal carriage |  | |

**Table S2**: The Detailed search strategy for Scopus, PubMed, Google scholar and Web of science Databases

| Database | Search Strategy |
| --- | --- |
| PubMed | ((((((((((((((((((((((((MRSA[Title/Abstract]) OR ("Methicillin-resistant Staphylococcus aureus"[Title/Abstract])) OR ("Methicillin resistant Staphylococcus aureus"[Title/Abstract])) OR ("Methicillin resistant S. aureus"[Title/Abstract])) OR ("Methicillin-resistant S. aureus"[Title/Abstract])) OR (MRSA infection[Title/Abstract])) OR ("Antibiotic-resistant S. aureus"[Title/Abstract])) OR ("Drug-resistant Staphylococcus aureus"[Title/Abstract])) OR (MRSA Disease*[Title/Abstract])) OR (MRSA Infection*[Title/Abstract])) AND (Nasal carriage[Title/Abstract])) OR (Nasopharyngeal coloniz*[Title/Abstract])) OR (Nasopharyngeal colonis*[Title/Abstract])) OR (Nasopharyngeal carriage[Title/Abstract])) AND (Human*[Title/Abstract])) OR (Patient*[Title/Abstract])) OR (Child*[Title/Abstract])) OR (Adult*[Title/Abstract])) OR (Individual*[Title/Abstract])) OR (Neonates[MeSH Terms])) OR (Pediatric[MeSH Terms])) OR (healthcare workers[Title/Abstract])) OR (Community member*[Title/Abstract])) AND (Ethiopia[Title/Abstract])) AND (("2015/01/01"[Date - Publication] : "2025/12/25"[Date - Publication])) |
| Scopus | (TITLE-ABS-KEY ("Methicillin-resistant Staphylococcus aureus" “MRSA” OR "Methicillin resistant S. aureus")) AND (TITLE-ABS-KEY (“Nasopharyngeal carriage” OR Nasopharyngeal colonis* OR Nasopharyngeal coloniz*)) AND (TITLE-ABS-KEY (Ethiopia)) AND (PUBYEAR >= 2000 AND PUBYEAR <= 2025) |
| Google scholar | Nasopharyngeal carriage rate, risk factors, and antimicrobial susceptibility pattern of methicillin-resistant *Staphylococcus aureus* in Ethiopia |
| Web of Science | Nasopharyngeal carriage rate, risk factors, and antimicrobial susceptibility pattern of methicillin-resistant *Staphylococcus aureus* in Ethiopia |

**Table S3: PRISMA_2020_abstract_checklist**

| **Section and Topic** | **Item #** | **Checklist item [1]** | **Reported (Yes/No)** |
| --- | --- | --- | --- |
| **TITLE** | | |  |
| Title | 1 | Identify the report as a systematic review. | Yes |
| **BACKGROUND** | | |  |
| Objectives | 2 | Provide an explicit statement of the main objective(s) or question(s) the review addresses. | Yes |
| **METHODS** | | |  |
| Eligibility criteria | 3 | Specify the inclusion and exclusion criteria for the review. | Yes |
| Information sources | 4 | Specify the information sources (e.g. databases, registers) used to identify studies and the date when each was last searched. | Yes |
| Risk of bias | 5 | Specify the methods used to assess risk of bias in the included studies. | Yes |
| Synthesis of results | 6 | Specify the methods used to present and synthesise results. | Yes |
| **RESULTS** | | |  |
| Included studies | 7 | Give the total number of included studies and participants and summarise relevant characteristics of studies. | Yes |
| Synthesis of results | 8 | Present results for main outcomes, preferably indicating the number of included studies and participants for each. If meta-analysis was done, report the summary estimate and confidence/credible interval. If comparing groups, indicate the direction of the effect (i.e. which group is favoured). | Yes |
| **DISCUSSION** | | |  |
| Limitations of evidence | 9 | Provide a brief summary of the limitations of the evidence included in the review (e.g. study risk of bias, inconsistency and imprecision). | Yes |
| Interpretation | 10 | Provide a general interpretation of the results and important implications. | Yes |
| **OTHER** | | |  |
| Funding | 11 | Specify the primary source of funding for the review. | Yes |
| Registration | 12 | Provide the register name and registration number. | Yes |

**Table S4: The 27-Item Checklist of the PRISMA Statement**

| **Section/topic** | **Item No** | **Checklist item** | **Reported on page No** |
| --- | --- | --- | --- |
| TITLE | | | |
| **Title** | 1 | Identify the report as a systematic review. | 1 |
| ABSTRACT | | | |
| **Abstract** | 2 | See the PRISMA 2020 for Abstracts checklist. | 2 and supplementary table presented above (**Table S3)** |
| INTRODUCTION | | | |
| **Rationale** | 3 | Describe the rationale for the review in the context of existing knowledge. | 3-4 |
| **Objectives** | 4 | Provide an explicit statement of the objective(s) or question(s) the review addresses. | 4 |
| METHODS | | | |
| **Eligibility criteria** | 5 | Specify the inclusion and exclusion criteria for the review and how studies were grouped for the syntheses. | 4-5 |
| **Information sources** | 6 | Specify all databases, registers, websites, organisations, reference lists and other sources searched or consulted to identify studies. Specify the date when each source was last searched or consulted. | 4 |
| **Search strategy** | 7 | Present the full search strategies for all databases, registers and websites, including any filters and limits used. | 4 and Supplementary Tables S1 and S2 presented above |
| **Selection process** | 8 | Specify the methods used to decide whether a study met the inclusion criteria of the review, including how many reviewers screened each record and each report retrieved, whether they worked independently, and if applicable, details of automation tools used in the process. | 4-5 |
| **Data collection process** | 9 | Specify the methods used to collect data from reports, including how many reviewers collected data from each report, whether they worked independently, any processes for obtaining or confirming data from study investigators, and if applicable, details of automation tools used in the process. | 5 |
| **Data items** | 10a | List and define all outcomes for which data were sought. Specify whether all results that were compatible with each outcome domain in each study were sought (e.g. for all measures, time points, analyses), and if not, the methods used to decide which results to collect. | 5 |
|  | 10b | List and define all other variables for which data were sought (e.g. participant and intervention characteristics, funding sources). Describe any assumptions made about any missing or unclear information. | 5 |
| **Study risk of bias assessment** | 11 | Specify the methods used to assess risk of bias in the included studies, including details of the tool(s) used, how many reviewers assessed each study and whether they worked independently, and if applicable, details of automation tools used in the process. | 5 and  **Table S5 (attached here below).** |
| **Effect measures** | 12 | Specify for each outcome the effect measure(s) (e.g. risk ratio, mean difference) used in the synthesis or presentation of results. | 5 |
| **Synthesis methods** | 13a | Describe the processes used to decide which studies were eligible for each synthesis (e.g. tabulating the study intervention characteristics and comparing against the planned groups for each synthesis (item #5)). | 5-6 |
|  | 13b | Describe any methods required to prepare the data for presentation or synthesis, such as handling of missing summary statistics, or data conversions. | 6-7 |
|  | 13c | Describe any methods used to tabulate or visually display results of individual studies and syntheses. | 6 |
|  | 13d | Describe any methods used to synthesize results and provide a rationale for the choice(s). If meta-analysis was performed, describe the model(s), method(s) to identify the presence and extent of statistical heterogeneity, and software package(s) used. | 6 |
|  | 13e | Describe any methods used to explore possible causes of heterogeneity among study results (e.g. subgroup analysis, meta-regression). | 6-7 |
|  | 13f | Describe any sensitivity analyses conducted to assess robustness of the synthesized results. | 6 |
| **Reporting bias assessment** | 14 | Describe any methods used to assess risk of bias due to missing results in a synthesis (arising from reporting biases). | 6 |
| **Certainty assessment** | 15 | Describe any methods used to assess certainty (or confidence) in the body of evidence for an outcome. | 6 |
| RESULTS | | | |
| **Study selection** | 16a | Describe the results of the search and selection process, from the number of records identified in the search to the number of studies included in the review, ideally using a flow diagram. | 7 and figure 1 |
|  | 16b | Cite studies that might appear to meet the inclusion criteria, but which were excluded, and explain why they were excluded. | **Not applicable** |
| **Study characteristics** | 17 | Cite each included study and present its characteristics. | **Table 1** |
| **Risk of bias in studies** | 18 | Present assessments of risk of bias for each included study. | 12 (Figure 4) |
| **Results of individual studies** | 19 | For all outcomes, present, for each study: (a) summary statistics for each group (where appropriate) and (b) an effect estimate and its precision (e.g. confidence/credible interval), ideally using structured tables or plots. | 8-9 and (Fig. 2) |
| **Results of syntheses** | 20a | For each synthesis, briefly summarise the characteristics and risk of bias among contributing studies. | Table 1 & Supplementary **Table S5.** |
|  | 20b | Present results of all statistical syntheses conducted. If meta-analysis was done, present for each the summary estimate and its precision (e.g. confidence/credible interval) and measures of statistical heterogeneity. If comparing groups, describe the direction of the effect. | 9 and figure 2 |
|  | 20c | Present results of all investigations of possible causes of heterogeneity among study results. | 10-12, Table 2 and 3 and figure 3 |
|  | 20d | Present results of all sensitivity analyses conducted to assess the robustness of the synthesized results. | 12 and figure 4 |
| **Reporting biases** | 21 | Present assessments of risk of bias due to missing results (arising from reporting biases) for each synthesis assessed. | 14 and figure 5 |
| **Certainty of evidence** | 22 | Present assessments of certainty (or confidence) in the body of evidence for each outcome assessed. | 12 |
| DISCUSSION | | | |
| **Discussion** | 23a | Provide a general interpretation of the results in the context of other evidence. | 20 |
|  | 23b | Discuss any limitations of the evidence included in the review. | 22 |
|  | 23c | Discuss any limitations of the review processes used. | 22 |
|  | 23d | Discuss implications of the results for practice, policy, and future research. | 20-22 |
| OTHER INFORMATION | | | |
| **Registration and protocol** | 24a | Provide registration information for the review, including register name and registration number, or state that the review was not registered. | **CRD420251047192** |
|  | 24b | Indicate where the review protocol can be accessed, or state that a protocol was not prepared. | From **PROSPERO** |
|  | 24c | Describe and explain any amendments to information provided at registration or in the protocol. | Not applicable |
| **Support** | 25 | Describe sources of financial or non-financial support for the review, and the role of the funders or sponsors in the review. | Not applicable |
| **Competing interests** | 26 | Declare any competing interests of review authors. | 23 |
| **Availability of data, code and other materials** | 27 | Report which of the following are publicly available and where they can be found: template data collection forms; data extracted from included studies; data used for all analyses; analytic code; any other materials used in the review. | 23 |

**Table S5: Critical appraisal of studies included in the systematic review and meta-analysis for pooled nasopharyngeal carriage rate, antibacterial susceptibility and risk factors associated with MRSA in Ethiopia, 2025.**

| **JBI Appraisal Checklist for cross-sectional Studies** | | | | | | | | | | | |
| --- | --- | --- | --- | --- | --- | --- | --- | --- | --- | --- | --- |
| Study ID | Q1 | Q2 | Q3 | Q4 | Q5 | Q6 | Q7 | Q8 | Q9 | Total | **Quality** |
| Abie, S., et al. [2] | Yes | Yes | Yes | Yes | Yes | Yes | Yes | Yes | NA | 8/9 | High |
| Abiye Tigabu, A. T., et al. [3] | Yes | Yes | Yes | Yes | Yes | Yes | Yes | Yes | NA | 8/9 | High |
| Adisu, M., et al. [4] | No | Yes | Yes | Yes | Yes | Yes | Yes | No | NA | 6/9 | Medium |
| Alemayehu Reta, A. R., et al. [5] | No | Yes | Yes | Yes | Yes | Yes | Yes | No | NA | 6/9 | Medium |
| Eba, W. W., et al. [6] | Yes | Yes | No | Yes | No | Yes | Yes | No | NA | 5/9 | Medium |
| Efa, F., et al. [7] | Yes | No | Yes | Yes | No | Yes | Yes | No | NA | 5/9 | Medium |
| Gebremedhn, G., et al. [8] | Yes | Yes | Yes | Yes | Yes | Yes | Yes | Yes | NA | 7/9 | High |
| Gebremeskel, F. T., et al. [9] | No | Yes | Yes | Yes | Yes | Yes | Yes | No | NA | 6/9 | Medium |
| Kahsay, A. G., et al. [10] | Yes | No | Yes | Yes | No | Yes | Yes | No | NA | 5/9 | Medium |
| Kejela, T. and F. Dekosa [11] | Yes | Yes | Yes | Yes | Yes | Yes | Yes | No | NA | 7/9 | High |
| Legese, H., et al. [12] | No | Yes | Yes | Yes | Yes | Yes | Yes | Yes | NA | 7/9 | High |
| Mamo, G., et al. [13] | No | Yes | Yes | Yes | Yes | Yes | Yes | No | NA | 6/9 | Medium |
| Manilal, A., et al. [14] | No | Yes | Yes | Yes | Yes | Yes | Yes | No | NA | 6/9 | Medium |
| Mekonnen, E. G., et al. [15] | No | Yes | Yes | Yes | Yes | Yes | Yes | Yes | NA | 7/9 | High |
| Mekuriya, E., et al. [16] | No | Yes | Yes | Yes | Yes | Yes | Yes | No | NA | 6/9 | Medium |
| Muhaba, H., et al. [17] | No | Yes | Yes | Yes | Yes | Yes | Yes | Yes | NA | 7/9 | High |
| Mulu, M., et al. [18] | No | Yes | Yes | Yes | Yes | Yes | Yes | No | NA | 6/9 | Medium |
| Shume, T., et al. [19] | No | Yes | Yes | Yes | Yes | Yes | Yes | No | NA | 6/9 | Medium |
| Weldegebreal, F., et al. [20] | No | No | Yes | Yes | Yes | Yes | Yes | No | NA | 5/9 | Medium |
| Yohannes Zenebe, Y. Z., et al. [21] | No | Yes | Yes | Yes | Yes | Yes | Yes | No | NA | 6/9 | Medium |

**Explanation of Prevalence Critical Appraisal questions [22]**

Answers: Yes, No, Unclear (UC) or Not/Applicable (NA)

1. **Was the sample frame appropriate to address the target population?**

This question relies upon knowledge of the ***broader characteristics of the population of interest and the geographical area.*** If the study is of women with breast cancer, knowledge of at least the characteristics, demographics and medical history is needed. The term “target population” should not be taken to infer every individual from everywhere or with similar disease or exposure characteristics. Instead, give consideration to specific population characteristics in the study, including age range, gender, morbidities, medications, and other potentially influential factors. For example, a sample frame may not be appropriate to address the target population if a certain group has been used (such as those working for one organization, or one profession) and the results then inferred to the target population (i.e. working adults). A sample frame may be appropriate when it includes almost all the members of the target population (i.e. a census, or a complete list of participants or complete registry data).

2. **Were study participants recruited in an appropriate way?**

Studies may report ***random sampling from a population*,** and the methods section should report how sampling was performed. Random probabilistic sampling from a defined subset of the population (sample frame) should be employed in most cases, however, random probabilistic sampling is not needed when everyone in the sampling frame will be included/ analysed. For example, reporting on all the data from a good census is appropriate as a good census will identify everybody. When using cluster sampling, such as a random sample of villages within a region, the methods need to be clearly stated as the precision of the final prevalence estimate incorporates the clustering effect. ***Convenience samples,*** such as a street survey or interviewing lots of people at public gatherings are not considered to provide a representative sample of the base population.

3. **Was the sample size adequate?**

The larger the sample, the narrower will be the confidence interval around the prevalence estimate, making the results more precise. An adequate sample size is important to ensure good precision of the final estimate. Ideally, we are looking for evidence that the authors conducted a sample size calculation to determine an adequate sample size. This will estimate how many subjects are needed to produce a reliable estimate of the measure(s) of interest. For conditions with a low prevalence, a larger sample size is needed. Also consider sample sizes for subgroup (or characteristics) analyses, and whether these are appropriate. Sometimes, the study will be large enough (as in large national surveys) whereby a sample size calculation is not required. In these cases, sample size can be considered adequate.

When there is no sample size calculation and it is not a large national survey, the reviewers may consider conducting their own sample size analysis using the following formula: (Naing et al. 2006, Daniel 1999)

$$n= \frac{Z^{2}P(1-P)}{d^{2}}$$

Where: n= sample size

Z = Z statistic for a level of confidence

P = Expected prevalence or proportion (in proportion of one; if 20%, P = 0.2) d = precision (in proportion of one; if 5%, d=0.05)

Ref:

Naing L, Winn T, Rusli BN. Practical issues in calculating the sample size for prevalence studies Archives of Orofacial Sciences. 2006; 1:9-14.

Daniel WW. Biostatistics: A Foundation for Analysis in the Health Sciences. Edition. 7th ed. New York: John Wiley & Sons. 1999.

**4. Were the study subjects and setting described in detail?**

Certain diseases or conditions vary in prevalence across different geographic regions and populations (e. g. Women vs. Men, sociodemographic variables between countries). The study sample should be described in sufficient detail so that other researchers can determine if it is comparable to the population of interest to them.

**5. Was data analysis conducted with sufficient coverage of the identified sample?**

***Coverage bias*** can occur when ***not all subgroups of the identified sample respond at the same rate***. For instance, you may have a very high response rate overall for your study, but the response rate for a certain subgroup (i.e. older adults) may be quite low.

**6. Were valid methods used for the identification of the condition?**

Here we are looking for ***measurement or classification bias.*** Many health problems are not easily diagnosed or defined and some measures may not be capable of including or excluding appropriate levels or stages of the health problem. If the outcomes were assessed based on existing definitions or ***diagnostic criteria***, then the answer to this question is likely to be yes. If the outcomes were assessed using observer reported, or self-reported scales, the risk of over- or under-reporting is increased, and objectivity is compromised. Importantly, determine if the measurement tools used were validated instruments as this has a significant impact on outcome assessment validity.

**7. Was the condition measured in a standard, reliable way for all participants?**

Considerable judgment is required to determine the presence of some health outcomes. Having established the validity of the outcome measurement instrument (see item 6 of this scale), it is important to establish how the measurement was conducted. Were those involved in ***collecting data trained or educated in the use of the instrument/s***? If there was more than one data collector, were they similar in terms of level of education, clinical or research experience, or level of responsibility in the piece of research being appraised? When there was more than one observer or collector, was there comparison of results from across the observers? Was the condition measured in the same way for all participants?

**8. Was there appropriate statistical analysis?**

Importantly, the numerator and denominator should be clearly reported, and ***percentages should be given with confidence intervals.*** The methods section should be detailed enough for reviewers to identify the analytical technique used and how specific variables were measured. Additionally, it is also important to assess the appropriateness of the analytical strategy in terms of the assumptions associated with the approach as differing methods of analysis are based on differing assumptions about the data and how it will respond.

**9. Was the response rate adequate, and if not, was the low response rate managed appropriately?**

A large number of dropouts, refusals or “not founds” amongst selected subjects may diminish a study’s validity, as can a low response rates for survey studies. The authors should clearly discuss the response rate and any reasons for non-response and compare persons in the study to those not in the study, particularly with regards to their socio-demographic characteristics. If reasons for non-response appear to be unrelated to the outcome measured and the characteristics of non-responders are comparable to those who do respond in the study (addressed in question 5, coverage bias), the researchers may be able to justify a more modest response rate.

**Reference**

1.	Page, M.J., J.E. McKenzie, P.M. Bossuyt, I. Boutron, T.C. Hoffmann, C.D. Mulrow, L. Shamseer, J.M. Tetzlaff, E.A. Akl, and S.E. Brennan, *The PRISMA 2020 statement: an updated guideline for reporting systematic reviews.* bmj, 2021. **372**.

2.	Abie, S., M. Tiruneh, and W. Abebe, *Methicillin-resistant Staphylococcus aureus nasal carriage among janitors working in hospital and non-hospital areas: a comparative cross-sectional study.* Annals of clinical microbiology and antimicrobials, 2020. **19**(1): p. 47.

3.	Tigabu, A., M. Tiruneh, and F. Mekonnen, *Nasal Carriage Rate, Antimicrobial Susceptibility Pattern, and Associated Factors of Staphylococcus aureus with Special Emphasis on MRSA among Urban and Rural Elementary School Children in Gondar, Northwest Ethiopia: A Comparative Cross‐Sectional Study.* Advances in preventive medicine, 2018. **2018**(1): p. 9364757.

4.	Adisu, M., S. Amankwah, R. Tamrat, Y. Alemu, T. Akeba, and M. Tadesse, *Colonization and associated risk factors of methicillin-resistant Staphylococcus aureus among people living with HIV at Jimma medical center, Southwest Ethiopia.* International Journal of Clinical and Experimental Medical Sciences, 2023. **9**(4): p. 65-74.

5.	Reta, A., L. Gedefaw, T. Sewunet, and G. Beyene, *Nasal carriage, risk factors and antimicrobial susceptibility pattern of methicillin resistant Staphylococcus aureus among school children in Ethiopia.* J Med Microb Diagn, 2015. **4**(1): p. 1-6.

6.	Eba, W.W., E. Zerihun, K. Terefe, and A.D. Demeke, *Nasal Methicillin-resistant Staphylococcus aureus Carriage, Its Multi-Drug Resistance Pattern and Associated Factors among Primary School Children At Chiro Town; Eastern Ethiopia.* 2025.

7.	Efa, F., Y. Alemu, G. Beyene, E.K. Gudina, and W. Kebede, *Methicillin-resistant Staphylococcus aureus carriage among medical students of Jimma University, Southwest Ethiopia.* Heliyon, 2019. **5**(1).

8.	Gebremedhn, G., T.T. Gebremariam, A.G. Wasihun, T.A. Dejene, and M. Saravanan, *Prevalence and risk factors of methicillin-resistant Staphylococcus aureus colonization among HIV patients in Mekelle, Northern Ethiopia.* Springerplus, 2016. **5**(1): p. 877.

9.	Gebremeskel, F.T., T. Alemayehu, and M.M. Ali, *Methicillin-resistant Staphylococcus aureus antibiotic susceptibility profile and associated factors among hospitalized patients at Hawassa University Comprehensive Specialized Hospital, Ethiopia.* IJID regions, 2022. **3**: p. 129-134.

10.	Kahsay, A.G., D.G. Hagos, G.K. Abay, and T.A. Mezgebo, *Prevalence and antimicrobial susceptibility patterns of methicillin-resistant Staphylococcus aureus among janitors of Mekelle University, North Ethiopia.* BMC research notes, 2018. **11**(1): p. 294.

11.	Kejela, T. and F. Dekosa, *High prevalence of MRSA and VRSA among inpatients of Mettu Karl referral hospital, southwest Ethiopia.* Tropical Medicine & International Health, 2022. **27**(8): p. 735-741.

12.	Legese, H., A.G. Kahsay, A. Kahsay, T. Araya, G. Adhanom, S. Muthupandian, and A. Gebreyesus, *Nasal carriage, risk factors and antimicrobial susceptibility pattern of methicillin resistant Staphylococcus aureus among healthcare workers in Adigrat and Wukro hospitals, Tigray, Northern Ethiopia.* BMC research notes, 2018. **11**(1): p. 250.

13.	Mamo, G., G. Ferede, and T.B. Eshete, *Nasal carriage rate, antimicrobial susceptibility patterns and associated risk factors of methicillin-resistant Staphylococcus aureus among prisoners in Dessie town, Northeast Ethiopia.* 2020.

14.	Manilal, A., M. Shewangizaw, M. Mama, T. Gezmu, and B. Merdekios, *Methicillin-resistant Staphylococcus aureus colonization in HIV patients of Arba Minch province, Ethiopia: carriage rates, antibiotic resistance, and biofilm formation.* Acta microbiologica et immunologica Hungarica, 2019. **66**(4): p. 469-483.

15.	Mekonnen, E.G., A. Birhanu, M. Yimer, S. Bizuneh, M. Gizachew, and B. Gelaw, *Colonization of methicillin-resistant Staphylococcus aureus and vancomycin-resistant enterococci and its associated factors in cancer patients at the university of Gondar comprehensive specialized hospital, Northwest Ethiopia.* Plos one, 2025. **20**(2): p. e0318242.

16.	Mekuriya, E., A. Manilal, A. Aklilu, M. Woldemariam, T. Hailu, and B. Wasihun, *Methicillin-resistant Staphylococcus aureus colonization among medicine and health science students, Arba Minch University, Ethiopia.* Scientific Reports, 2022. **12**(1): p. 10161.

17.	Muhaba, H., G.M. Fenta, and D. Gebretsadik, *Methicillin resistance Staphylococcus aureus nasal carriage and its associated factors among HIV patients attending art clinic at Dessie comprehensive specialized hospital, Dessie, North East Ethiopia.* PLOS Global Public Health, 2022. **2**(9): p. e0000838.

18.	Mulu, M., A. Getaneh, W. Ferede, and A. Gelaw, *Nasal carriage rate of methicillin resistant Staphylococcus Aureus, antimicrobial susceptibility pattern and associated risk factors among pregnant women at the university of Gondar comprehensive specialized hospital northwest, Ethiopia.* Ethiopian Journal of Health and Biomedical Sciences, 2021. **11**(1): p. 45-53.

19.	Shume, T., K. Urgesa, S. Mekonnen, F. Ayele, T. Tesfa, F. Tebeje, W. Wolde, M. Ahmed, K. Bogale, and S. Debebe, *Nasal carriage of MRSA among clinically affiliated undergraduate students at the College of Health and Medical Sciences, Haramaya University, Ethiopia.* Scientific reports, 2024. **14**(1): p. 29977.

20.	Weldegebreal, F., K. Urgesa, F. Ayele, K. Bogale, T. Shume, M. Ahmed, S. Debebe, F. Tebeje, H. Asmerom, and T. Tesfa, *Nasal carriage rate, associated factors, and antimicrobial susceptibility patterns of methicillin resistance Staphylococcus aureus among pre-clinical undergraduate students at the College of Health and Medical Sciences, Haramaya University, Ethiopia.* Frontiers in Public Health, 2024. **12**: p. 1354461.

21.	Zenebe, Y., M. Tibebu, B. Tulu, D. Mekonnen, and Z. Mekonnen, *Methicillin-resistant Staphylococcus aureus with genotyping method among human immunodeficiency virus positive pediatric patients in Northwest Ethiopia: A cross-sectional study design.* The Ethiopian Journal of Health Development, 2018. **32**(3).

22.	Munn, Z., S. Moola, K. Lisy, D. Riitano, and C. Tufanaru, *Methodological guidance for systematic reviews of observational epidemiological studies reporting prevalence and cumulative incidence data.* JBI Evidence Implementation, 2015. **13**(3): p. 147-153.
